# MFM-300 as High-Performance Sorbents for Water-Adsorption-Driven Cooling

**DOI:** 10.1021/jacs.4c16752

**Published:** 2025-04-08

**Authors:** Xue Han, Yinlin Chen, Jiangnan Li, Wanpeng Lu, Wenyuan Huang, Yuanjun Wang, Guixiang Wang, Ivan da Silva, Yongqiang Cheng, Luke L. Daemen, Pascal Manuel, Anibal J. Ramirez-Cuesta, Daniel Lee, Sihai Yang, Martin Schröder

**Affiliations:** † College of Chemistry, Beijing Normal University, Beijing 100091, China; ‡ Department of Chemistry, University of Manchester, Manchester M13 9PL, U.K.; § College of Chemistry and Molecular Engineering, Beijing National Laboratory for Molecular Sciences, Peking University, Beijing 100871, China; ∥ ISIS Facility, Rutherford Appleton Laboratory, Chilton OX11 0QX, U.K.; ⊥ Neutron Scattering Division, Neutron Sciences Directorate, Oak Ridge National Laboratory, Oak Ridge, Tennessee 37831, United States; # Department of Chemical Engineering, University of Manchester, Manchester M13 9PL, U.K.

## Abstract

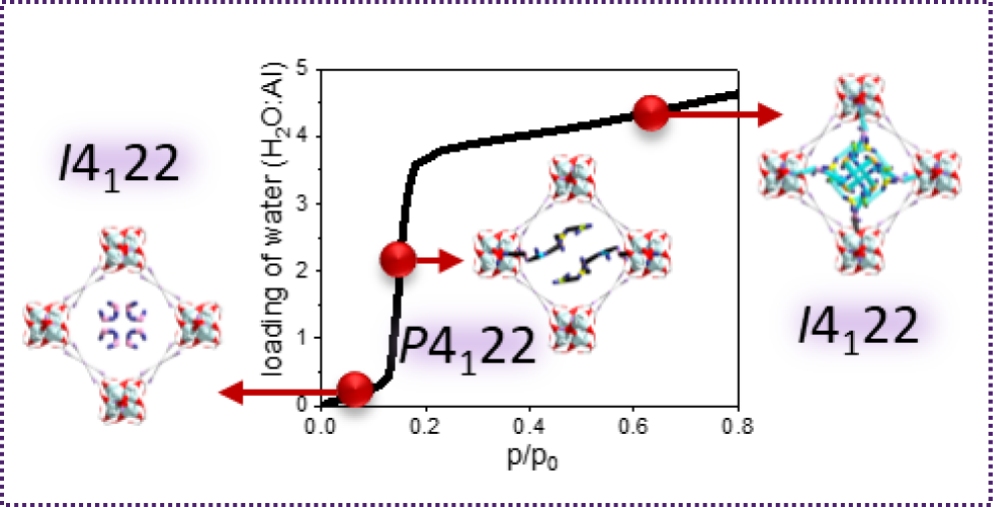

Adsorption-driven heat transfer is potentially a sustainable technology to decarbonize heating and cooling. However, the development of high-performance adsorbent–adsorbate working pairs remains extremely challenging. Here, we report a metal–organic framework/water working pair that can operate at an ultralow driving temperature (62 °C), showing a high coefficient of performance (COP) of 0.8 for cooling. The desirable features of MFM-300(M) (M = Al, Fe, Cr, V) for water adsorption have been elucidated by combined crystallographic and spectroscopic techniques. *In situ* neutron powder diffraction reveals the structural evolution of the MFM-300-D_2_O system via direct observation of the location of D_2_O at different stages of adsorption. Host–guest binding dynamics have been interrogated by *in situ* solid-state nuclear magnetic resonance spectroscopy and inelastic neutron scattering combined with modeling. This system promotes the use of renewable low-grade thermal energy rather than electricity to drive cooling.

## Introduction

Globally, the use of air conditioners accounts for nearly 20% of the total electricity used in buildings, and this trend is set to grow rapidly.^1^ Air conditioners use electric compressors to drive the transfer of heat through the compression and expansion of chlorofluorocarbon (CFC) refrigerants. In response to recent international agreements to tackle climate change, emphasis has been placed on reducing the upstream carbon intensity of electric air conditioners and phasing out CFC refrigerants that have high global warming potential. Adsorption-driven cooling (ADC) is considered an appealing technology because it is driven by low-grade thermal energy instead of electricity and can use water as the working fluid rather than CFCs. Currently, a few commercial ADC devices are available on the market, with others being developed at laboratory scale.^2^

**Scheme 1:**
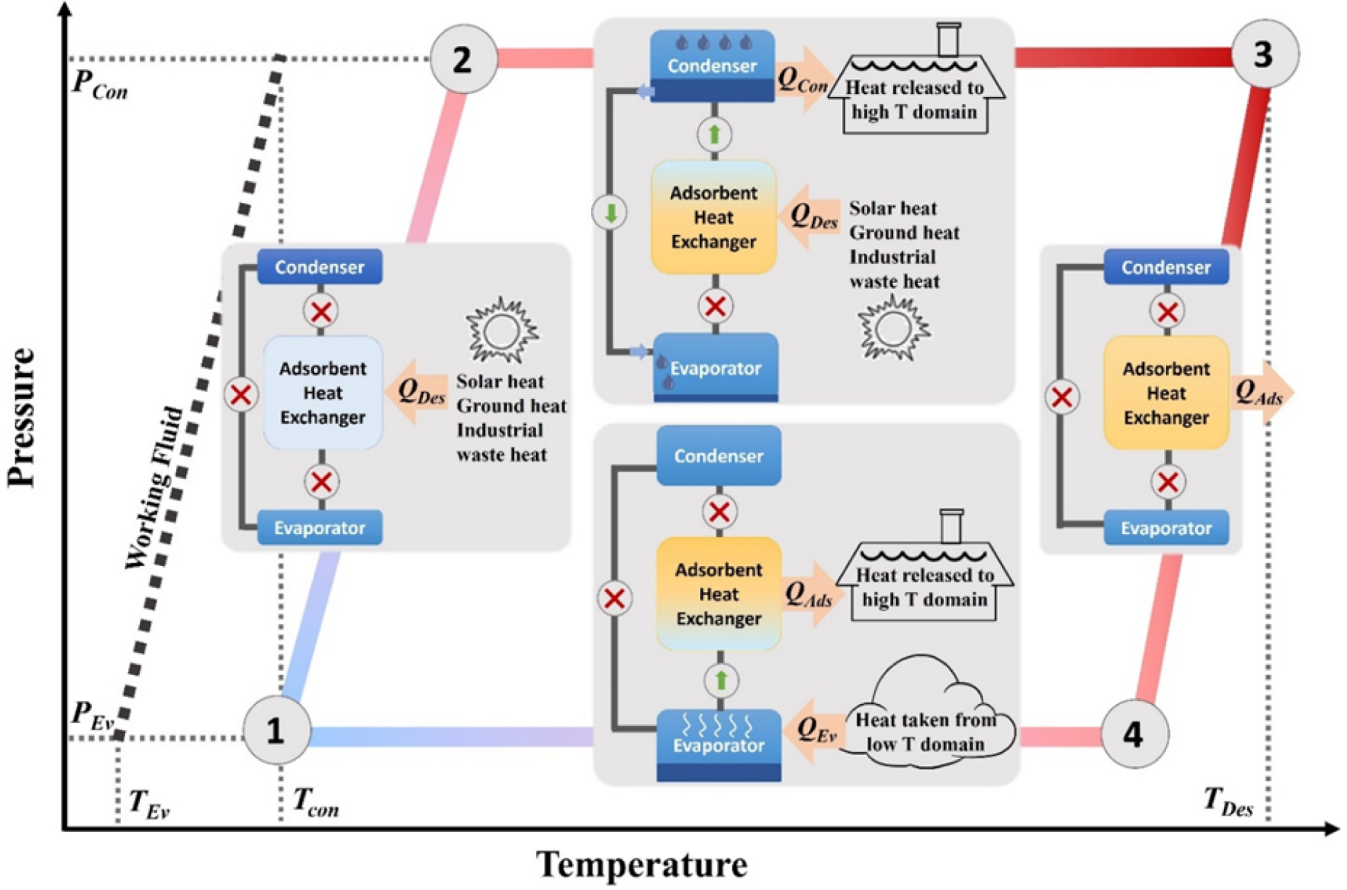
Diagram of an Isosteric Adsorption-Driven Heat Transfer Cycle Adsorption-triggered evaporation can extract heat from the surroundings to achieve cooling effects. The energy input (e.g., low-grade thermal energy) desorbs the working fluid from the sorbents.

Although the precise configurations of these devices vary, their underlying working principles are to extract heat from a low-temperature domain and release it to a high-temperature domain. This process can operate through a four-step cycle (Scheme 1), where the thermal effects associated with evaporation, adsorption, desorption, and condensation are exploited to achieve heat transfer. Based on this principle, the key to high-performance ADC hinges on developing effective sorbent materials that are highly stable, possess high working capacity, and can be regenerated at low temperatures, thus allowing low-grade heat to be used as the driving energy for such devices. Nearly all commercial ADC devices use silica gels and/or zeolites as sorbents due to their low production costs and high stability, but they also show clear drawbacks such as limited working capacity and high regeneration temperatures .^3^ Metal–organic frameworks (MOFs) represent a versatile family of porous materials that are highly designable in terms of framework topology, porosity, hydrophilicity, and chemical functionality. This has generated much interest in the use of MOFs as sorbents of water for a wide range of applications, such as water harvesting from air,^4,5^ humidity control^6^ and adsorption-driven heat applications.^7−10^ A few MOFs have demonstrated higher working capacity and/or higher coefficient of performance (COP) with low regeneration temperatures compared to commercial silica gel and SAPO-34 zeolites,^11^ but the development of more effective sorbents for ADC is urgently required.

We have previously reported that MFM-300 materials can successfully capture and release corrosive gases such as NO_2_, SO_2_ and NH_3_ reversibly. This is due to therobust “wine-rack” framework structure with hydroxyl-decorated channels that can anchor these substrates through optimal hydrogen bonding interactions, resulting in highly reversible adsorption.^12−15^ Encouraged by recent studies on the long-term stability of MFM-300(Al) where the MOF retained its crystallinity after being in contact with corrosive gases or water vapor for ca. 4 years,^16^ we report herein the development of MFM-300(M) (M = Al, Fe, Cr, V) as reversible sorbents of water for adsorption-driven cooling. Both high working capacities and COPs have been achieved, comparing favorably with commercial sorbents and other leading MOFs. A regeneration temperature as low as 62 °C is sufficient to regenerate the materials to reach a COP of >0.8 for MFM-300(M) (M = Al, Fe, Cr). The binding domains for adsorbed water molecules and hydrogen bonding networks in these materials have been visualized directly by *in situ* neutron powder diffraction (NPD) allowing direct observation of intermolecular hydrogen bonds as a function of the degree of hydration. *In situ* inelastic neutron scattering (INS), coupled with modeling, and solid-state nuclear magnetic resonance (ssNMR) spectroscopy afford key insights into the binding dynamics of MFM-300-H_2_O. This study builds opportunities for versatile low-grade heat sources to be utilized for cooling using MOF-driven ADC devices.

## Experimental Section

### Measurement of Isotherms

Adsorption isotherms for MFM-300(M) (M = Al, Fe, Cr, V) were measured using an Autosorb iQ Gas Sorption Analyzer based on a volumetric configuration. All samples were activated at 453 K under dynamic vacuum for 24 h before measurement, and the same batch of samples was reactivated before measuring the next isotherm at a different temperature. Cyclic adsorption/desorption of water with MFM-300(M) (*M* = Al, Fe) was conducted on a DVS Vacuum (Surface Measurement Systems Ltd., UK), a gravimetric vapor sorption instrument.

### Neutron Powder Diffraction

Structural determination of water-loaded MFM-300(M) (M = Al, Cr) was conducted at WISH, a long-wavelength powder and single-crystal neutron diffractometer at the ISIS Neutron and Muon Facility at Rutherford Appleton Laboratory (UK). The instrument utilizes a solid methane moderator, providing a high flux of cold neutrons with a large bandwidth, which is transported to the sample via an elliptical guide. Desolvated MFM-300(M) and a known amount of deuterated water were loaded into a cylindrical vanadium sample container and sealed with an indium vacuum seal. The cell was kept at 353 K for 24 h to allow complete dispersion of the water within the solid. Data collection was performed at 298 K. Rietveld refinements of the NPD patterns were performed by using the TOPAS software package. Additional details on the refinements are given in the Supporting Information.

### Inelastic Neutron Scattering and DFT Calculations

INS spectra were collected on the VISION spectrometer at the Spallation Neutron Source, Oak Ridge National Laboratory (USA). The samples of water-loaded MFM-300(M) were prepared in the same way as those used for NPD experiments. The temperature during data collection was controlled by using a closed-cycle refrigerator (CCR) cryostat (10 ± 0.1 K). Background spectra (sample can plus bare MFM-300) were subtracted to obtain difference spectra. Computational details for DFT calculations are described in SI Section 7.

### Solid-State Nuclear Magnetic Resonance Spectroscopy

ssNMR spectra were recorded on a Bruker 9.4 T (400 MHz ^1^H Larmor frequency) AVANCE III spectrometer equipped with a 4 mm HFX MAS probe. MFM-300(Al) was activated at 453 K under dynamic vacuum for 24 h and packed into a 4 mm o.d. zirconia rotor under inert conditions, sealed with a Kel-F rotor cap. After NMR measurements on the pristine MOF, the rotor was opened under an inert atmosphere, and the MOF was saturated with D_2_O before brief degassing to remove excess D_2_O; the rotor cap was then replaced. Experiments were conducted at ambient temperature under static conditions (for ^2^H NMR) or by using a MAS frequency of 12 kHz (for ^1^H, ^13^C, and ^27^Al NMR).

## Results and Discussion

### Adsorption Isotherms

**Figure 1:**
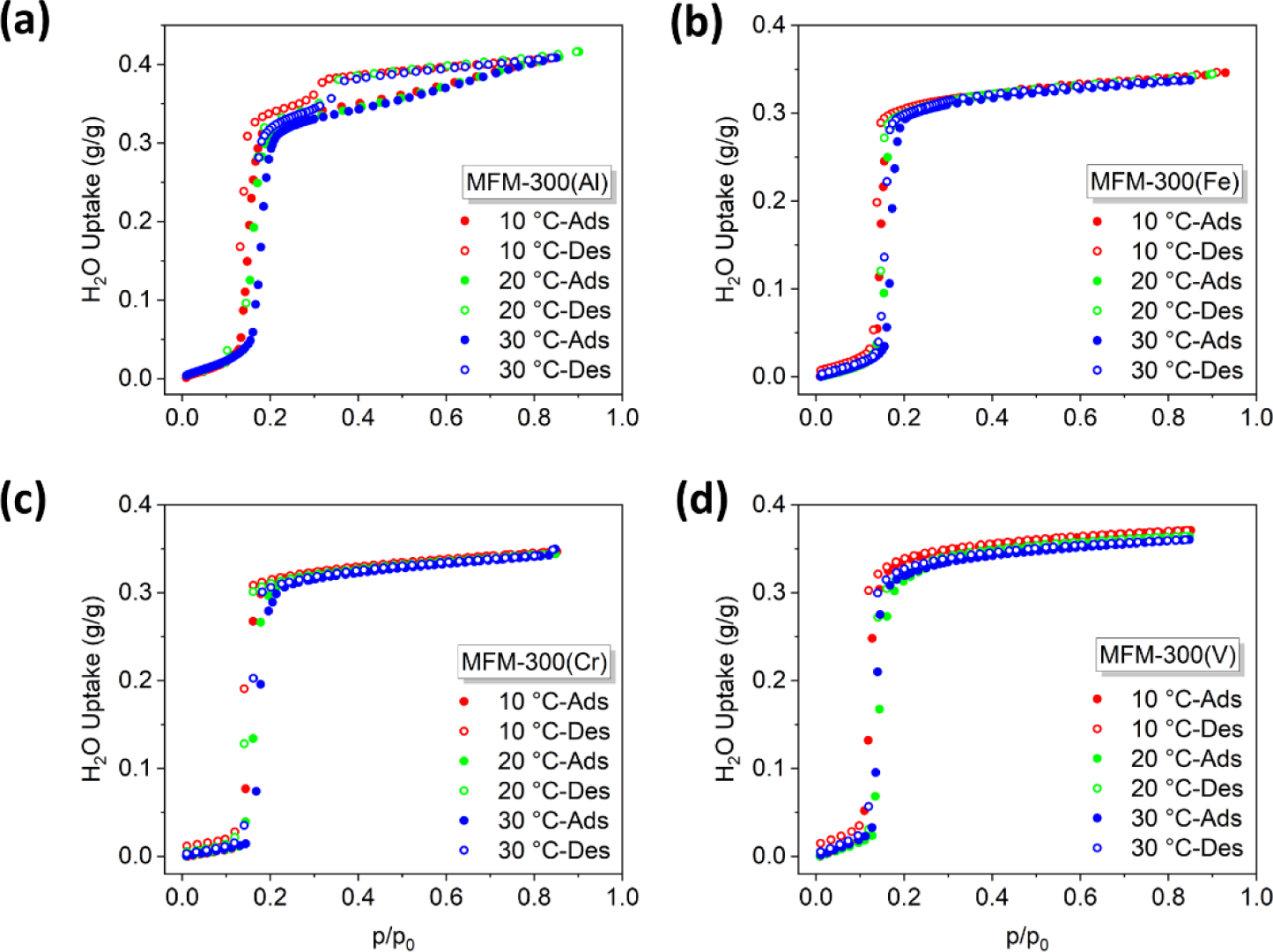
Adsorption isotherms of water in desolvated MFM-300(M) (M = Al, Fe, Cr, V) at 10, 20, and 30 °C at partial water vapor pressure (*P*/*P*_0_) between 0 and 0.9. (a) MFM-300(Al); (b) MFM-300(Fe); (c) MFM-300(Cr); (d) MFM-300(V).

Adsorption isotherms are the most convenient method for evaluating the intrinsic properties of a given sorbent. Commonly predefined temperature boundaries for the application of porous solids in ADC require *T*_ev_ = 5 °C, *T*_con_ = 30 °C for refrigeration, and *T*_ev_ = 10 °C, *T*_con_ = 30 °C for air-conditioning (ev = evaporation, con = condensation). An ideal adsorption isotherm should exhibit a single steep adsorption profile with a high uptake at *P*/*P*_0_ > 0.2.^7^ The adsorption isotherms of water for MFM-300(M) (M = Al, Fe, Cr, V) were measured at 10–30 °C, and uptakes of water reached 0.33, 0.31, 0.32, and 0.34 g·g^–1^, respectively, at 30 °C and *P*/*P*_0_ = 0.3 (Figure 1). These uptakes compare favorably not only to those of benchmark sorbents (SAPO-34: 0.29 g. g^–1^;^17^ and AQSOA-Z01, 02, 05: 0.22, 0.29, 0.23 g.g^–1^ respectively)^18^ but also to most reported leading MOFs that have been studied for adsorption-driven heat allocation [MIL-160: 0.38 g.g^–1^ (30 °C and *P*/*P*_0_ = 0.9);^11^ CAU-10: 0.35 g.g^–1^ (25 °C and *P*/*P*_0_ = 0.9);^11,19,20^ CAU-23: 0.37 g.g^–1^ (25 °C and *P*/*P*_0_ = 0.33);^21^ Co-CUK-1: 0.28 g.g^–1^ (30 °C and *P*/*P*_0_ = 0.4);^22^ MOF-303: 0.39 g.g^–1^ (30 °C and *P*/*P*_0_ = 0.3);^23^ MIP-200: 0.39 g.g^–1^ (30 °C and *P*/*P*_0_ = 0.3);^24^ KMF-1: 0.41 g.g^–1^ (25 °C and *P*/*P*_0_ = 0.33);^25^ KMF-2: 0.35 g.g^–1^ (30 °C and *P*/*P*_0_ = 0.2);^26^ Al-Fumarate: 0.32 g.g^–1^ (30 °C and *P*/*P*_0_ = 0.48);^27^ DUT-67: 0.32 g.g^–1^ (25 °C and *P*/*P*_0_ = 0.55);^28^ EMM-8: 0.24 g.g^–1^ (25 °C, *P*/*P*_0_ = 0.2)].^29^ A detailed comparison of benchmark MOFs for water adsorption has been summarized in several reports.^26,29,30^ All isotherms of MFM-300 show a single step at *P*/*P*_0_ = ∼0.2 and the adsorption/desorption cycles are fully reversible, confirming the feasibility of these MOFs to cycle between their maximum and minimum uptakes of water within a small window of *P*/*P*_0_. Fittings of the isotherms at three temperatures using the Clausius–Clapeyron equation^31^ give moderate values of heats of adsorption at around 49 kJ mol^–1^ for MFM-300, suggesting the facile regeneration of these materials, which is a key feature for their practical applications (Figures S1–S8).

### Thermodynamic Performance of the MFM-300/H_2_O Working Pair

The heat-transfer process in a MOF/water working pair can be viewed as a four-step cycle from a thermodynamic perspective, as illustrated in Scheme 1. At point ①, the MOF sorbent is at the lowest temperature with a maximum uptake of water; at point ③, the MOF sorbent is at the highest temperature with a minimum uptake of water. Thus, processes ①→② (isosteric heating) and ②→③ (isobaric desorption) can be viewed as the regeneration of the MOF, where thermal energy is taken up from the heat source (*Q*_des_) to desorb water. The water is subsequently condensed, releasing heat (*Q*_con_) to a high-temperature domain. ③→④ (isosteric cooling) and ④→① (isobaric adsorption) can be viewed as the heat-relocation step, where heat is taken up from the low-temperature domain through the evaporation of water (*Q*_ev_) at the evaporator. Continuous evaporation is maintained by the spontaneous adsorption of water into the MOF at the sorbent bed, where heat (*Q*_ads_) is released to the high-temperature domain because adsorption is an exothermic process. This cycle can thus be operated as a heat pump to produce heat at the low-temperature domain using *Q*_con_ and *Q*_ads_, or to achieve cooling by removing heat from the low-temperature domain by making use of *Q*_ev_. The working capacity (Δ*W*) is defined as the usable uptake of water between points ① and ③ of the cycle, and the COP for ADC is defined as the useful energy output (*Q*_ev_) that is withdrawn by the evaporator divided by the energy required as an input (*Q*_des_) to desorb water from the MOF.

**Figure 2:**
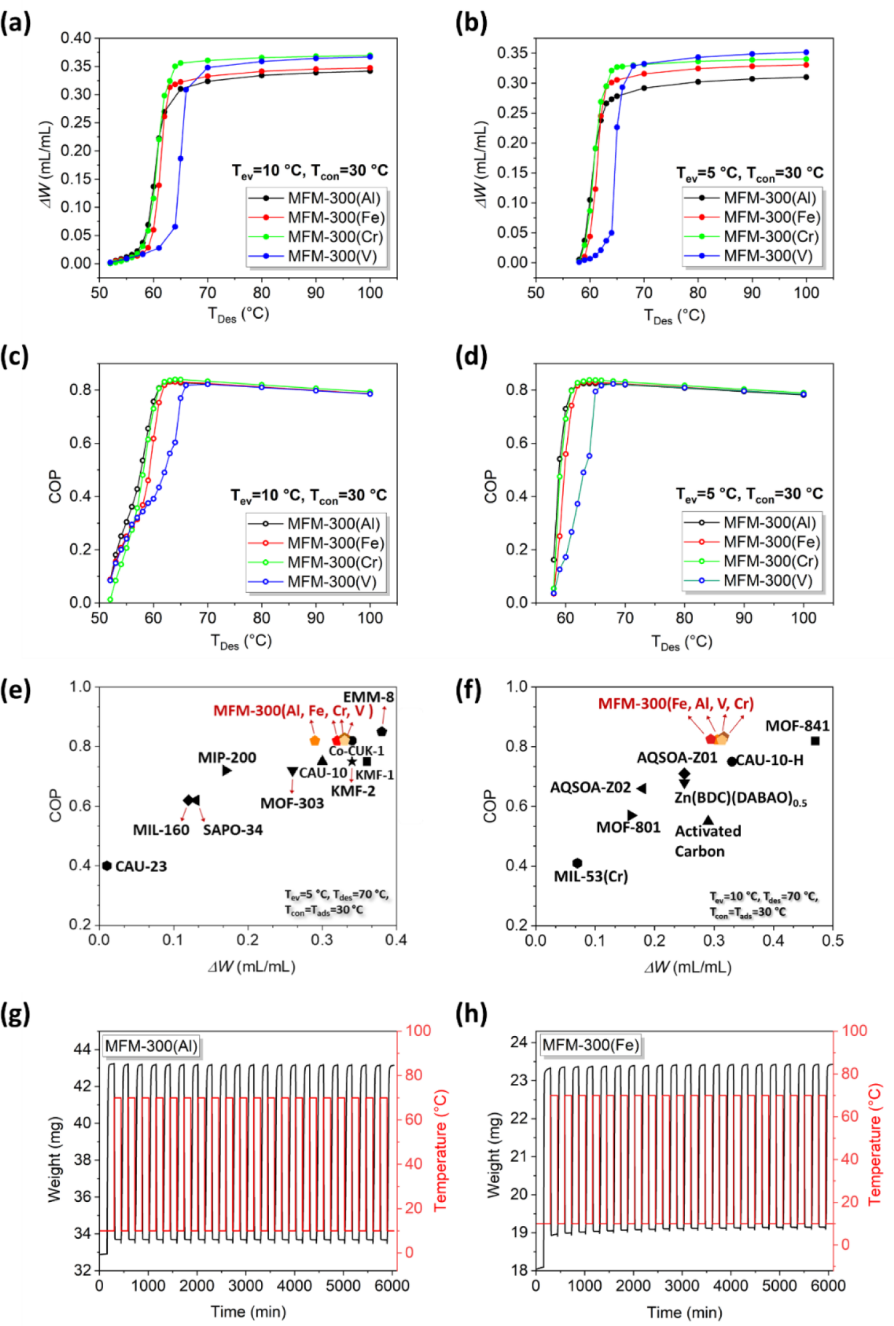
Analysis of the performance of MFM-300 for heat transfer. (a,b) Working capacities and (c,d) COP for MFM-300(M) (M = Al, Fe, Cr, V)/water working pairs for adsorption-driven cooling as a function of desorption temperature (*T*_des_). These calculations were performed with temperature boundaries set at *T*_ev_ = 5 °C, *T*_con_ = *T*_ads_ = 30 °C and *T*_ev_ = 10 °C, T_con_ = *T*_ads_ = 30 °C. Comparison of COP versus ΔW for MFM-300(M) (M = Al, Fe Cr, V) and reported porous sorbents with temperature boundaries set at (e) *T*_ev_ = 5 °C, *T*_con_ = *T*_ads_ = 30 °C, and *T*_des_ = 70 °C; and (f) of *T*_ev_ = 10 °C, *T*_con_ = *T*_ads_ = 30 °C, and *T*_des_ = 70 °C. Thermogravimetric profiles of (g) MFM-300(Al) and (h) MFM-300(Fe) under multiple cycles of adsorption (10 °C, R.H. = 30%) and desorption (70 °C, R.H. = 10%).

The values for working capacity (Δ*W)* and coefficient of performance (COP) for MFM-300(M) (M = Al, Fe, Cr, V) for ADC have been calculated from the adsorption isotherms using standard thermodynamic models with two sets of commonly applied temperature boundaries (*T*_ev_ = 5 °C, *T*_con_ = 30 °C) for refrigeration and (*T*_ev_ = 10 °C, *T*_con_ = 30 °C) for air-conditioning.^7^ A driving temperature (*T*_des_) below 90 °C is recognized as sufficiently low for a sorbent to use waste heat to achieve cooling.^29^ As shown in Figure 2a, for air-conditioning, MFM-300(M) (M = Al, Fe, Cr) affords high values for Δ*W* of 0.31, 0.32, and 0.35 mL.mL^–1^, respectively, at an ultralow driving temperature (*T*_des_) of 64 °C, which is one of the lowest reported values of *T*_des_ for MOFs.^21^ For MFM-300(V), a *T*_des_ of 68 °C is required to maintain a high value for Δ*W* of 0.33 mL.mL^–1^, which is still sufficiently low for the utilization of low-grade waste heat from various industrial processes.^32^ Similarly, for refrigeration (Figure 2b), high Δ*W* values of 0.27, 0.30, and 0.31 mL.mL^–1^ can be achieved by MFM-300(M) (M = Al, Fe, Cr) at *T*_des_ = 64 °C, and for MFM-300(V), Δ*W* reaches 0.33 mL.mL^–1^ with *T*_des_ at 70 °C. The calculated COPs for ADC are shown in Figure 2c,d, and with both sets of *T*_ev_ and *T*_con_, high values for COP of 0.82 can be achieved with *T*_des_ as low as 62 °C for MFM-300(M) (M = Al, Fe, Cr); for MFM-300(V), a value of *T*_des_ = 66 °C is required to reach a COP of 0.82. It is worth noting that with both sets of temperature boundaries, for refrigeration (Figure 2e) and air-conditioning (Figure 2f), the materials MFM-300(M) are among the best-performing porous sorbents such as MIL-160,^11^ CAU-10,^24^ MOF-801,^24^ MIP-200,^24^ CAU-10-pydc,^33^ Co-CUK-1,^22^ MOF-303,^25^ KMF-1,^25^ KMF-2,^26^ EMM-8,^29^ and Zr-adip^34^ in terms of affording both high Δ*W* and COP under low regeneration temperature conditions. Encouraged by these results and considering that MFM-300(Fe) can be produced readily at a kilogram scale,^35^ the stability of MFM-300(Fe) and MFM-300(Al) was tested further under aqueous and working conditions. As confirmed by powder X-ray diffraction (PXRD) and adsorption isotherms (Figures S10–S12), MFM-300(Al) and MFM-300(Fe) retain their crystallinity and adsorption capacity of water after being soaked in water at room temperature for one month or in boiling water for 5 days. The stabilities of MFM-300(Al) and MFM-300(Fe) over multiple cycles are further confirmed by the thermogravimetric profiles over 20 cycles of adsorption [10 °C, relative humidity (RH) = 30%] and desorption (70 °C, RH = 10%), where the adsorption capacity is fully retained (Figure 2g,h). Thus, these results demonstrate the feasibility of applying the MFM-300/water working pairs for adsorption-driven heat transfer.

**Figure 3:**
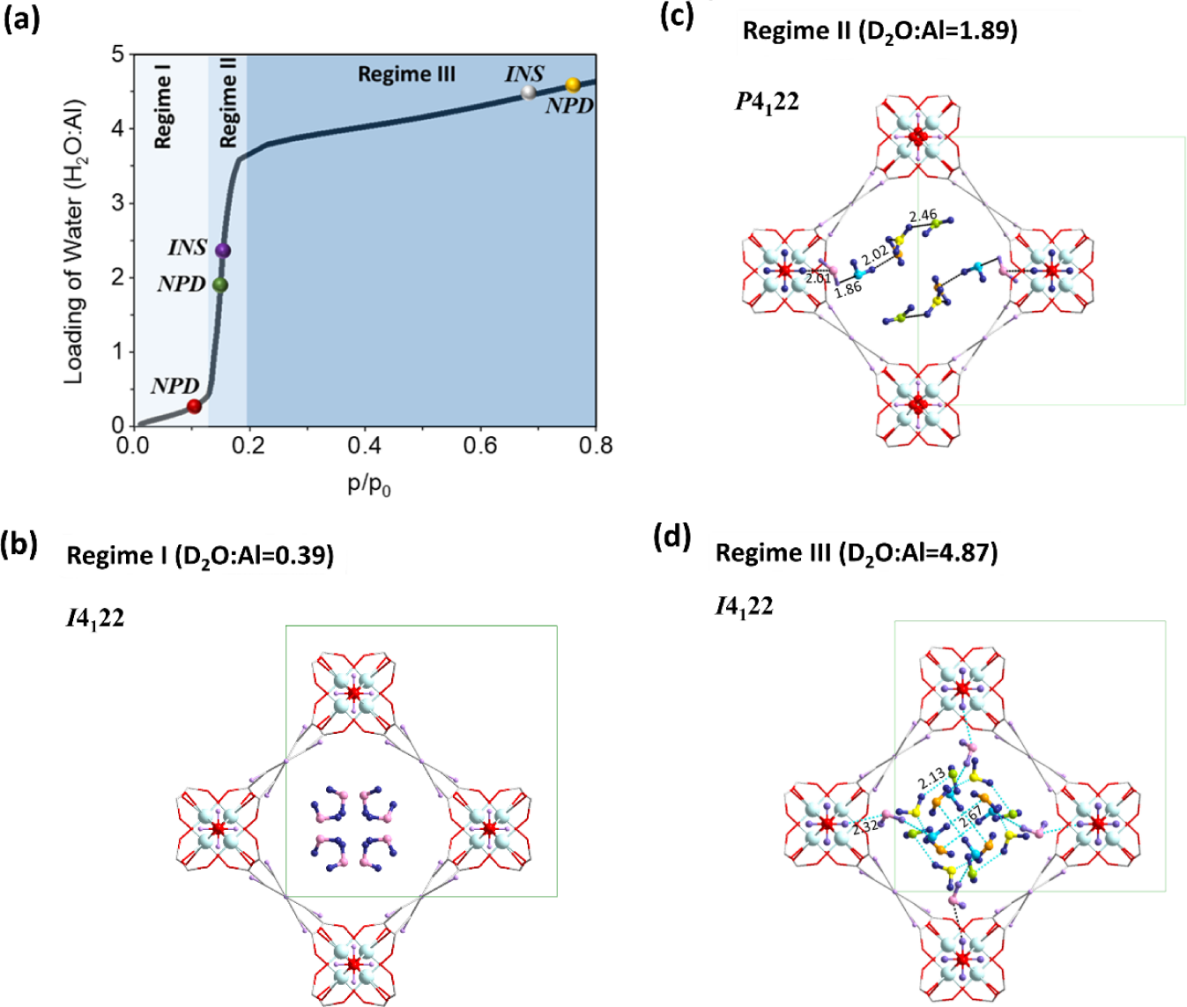
(a) Adsorption profile for the loading of water into MFM-300(Al) at 293 K and the corresponding reported NPD and INS analyses. Views of the structure of (b) [AlC_8_H_4_O_5_]·0.39(D_2_O), (c) [AlC_8_H_4_O_5_]·1.89(D_2_O), and (d) [AlC_8_H_4_O_5_]·4.87(D_2_O). All structures were determined from Rietveld refinements of the NPD data.

### Mechanism of Adsorption

The mechanism of adsorption of water in MOFs has been studied extensively, and several structural features have been found to play an important role in their water adsorption properties. For example, MOFs with coordinatively unsaturated metal nodes, such as HKUST-1,^36^ tend to bind water molecules directly to the metal ion, and the desorption of these bound water molecules usually leads to the collapse of the framework. MOFs with pore diameters greater than 2 nm, such as MIL-101(Cr),^37^ often exhibit stepwise isotherms showing hysteresis due to capillary condensation. For many microporous MOFs, the saturated water uptake is often positively correlated with the pore volume and BET surface area of the materials; on the other hand, the inflection points and the steepness of the isotherms can be tuned by many strategies, such as ligand functionality,^38^ linker installation^39^ and metal oxide chain twisting.^40^ Here, the molecular basis for the excellent adsorption properties of MFM-300(M) (i.e., single steep step in the adsorption profile, high saturation uptake, and little hysteresis) has been investigated by a combination of *in situ* NPD, ssNMR, and INS, combined with density functional theory (DFT) calculations.

The water adsorption isotherms for MFM-300(M) exhibit a favorable single step at low pressure (*P*/*P*_0_ = ∼0.2), indicating the rapid formation of water clusters within the pores. The relative invisibility of protons in X-ray diffraction experiments naturally places uncertainties in the analysis of the hydrogen bonding network of water clusters within MOFs. Here, NPD was applied to unambiguously determine the position of adsorbed D_2_O molecules, including the position of deuterium, at each regime of the adsorption isotherm for MFM-300(Al) and MFM-300(Cr) (Figures 3 and S14). In Regime I, the uptake of water increases only slightly (from D_2_O:Al = 0 to 0.5) with increasing pressure from *P*/*P*_0_ = 0 to 0.12. This is due to the hydrophobic nature of the bulk framework as a result of the aromatic linkers and the absence of strong binding sites for water, such as coordinatively unsaturated metal nodes.^41,42^ Upon increasing the loading of water to D_2_O:Al = 0.39, one crystallographic site for water located near the center of the pore was observed without any apparent interaction with the framework (Figures 3b). This is somewhat surprising given that all previously studied small guest molecules (e.g., NH_3_,^13^ H_2_S,^43^ CH_4_,^44^ H_2_,^44^ CO_2_,^45^ SO_2_,^45^ NO_2_,^15^ hydrocarbons,^46^ when adsorbed in MFM-300, are preferentially anchored at the corner of the pore by the bridging hydroxyl groups within the [M(OH)_2_O_4_]_∞_ chain. Also, in reported MOFs incorporating M–OH moieties, adsorbed water molecules are observed to bind primarily to the M–OH sites.^47^ Interestingly, in a recent study of MOF-303 for water harvesting, the first water molecule was found to strongly bind to the pyrazole group of the organic linkers and the μ_2_–OH group of the metal oxide chains.^38^ The reason for the binding of water being distinct in MFM-300 is likely due to the high dipole moment of water (1.87 D), and thus the hydrophobicity of the nonpolar pore interior offsets the dipole–dipole attraction from the hydroxyl sites. Indeed, the lack of such direct interaction between M–OH groups and water molecules rationalizes the presence of Regime I in the adsorption isotherm; other guest molecules that bind to the M–OH groups at low loadings display isotherms that show immediate increases in uptake at low partial pressures. It is worth noting that the presence of Regime I, as opposed to a typical type-I isotherm, is crucial in ensuring a sufficiently low temperature for desorption, which is one of the key criteria required to use low-grade thermal energy to drive heat transfer in the ADC.

As the pressure increases into Regime II (*P*/*P*_0_ = 0.12–0.20), a sharp increase in water uptake is observed from D_2_O:Al = 0.5–3.8, indicating pore filling and the formation of water chains and clusters. At a loading of D_2_O:Al = 1.89, five sites are revealed by NPD, with one site showing hydrogen bonding interactions with the bridging hydroxyl groups (H_hydroxyl_···O_D2O_ = 2.01 Å); the other four sites interact with adjacent water molecules through hydrogen bonding interactions (D_D2O_···O_D2O_ = 1.86–2.46 Å) (Figures 3c). In Regime III, with D_2_O:Al = 4.87, a complete filling of the pore by the formation of water clusters has occurred (Figures 3d). For both structures within Regimes II and III (D_2_O:Al = 1.89 and 4.87, respectively), hydrogen–deuterium exchange is observed between the adsorbed D_2_O molecules and the bridging −OH groups, whereas significantly, no such exchange is seen in Regime I (D_2_O:Al = 0.39). This further confirms that direct host–guest interaction can only be induced when the vapor pressure of water reaches a critical threshold to surmount the hydrophobic repulsion of the framework.^48^ It is also worth noting that when stepping into Regime II, the emergence of hydrogen bonding between the adsorbed water and the bridging hydroxyl not only leads to the sharp increase in uptake but also induces deformation of the framework, as evidenced by the change in space group from *I*4_1_22 for [AlC_8_H_4_O_5_]·0.39(D_2_O) to *P*4_1_22 for [AlC_8_H_4_O_5_]·1.89(D_2_O). Interestingly, in Regime III, upon saturation of the adsorption, the structure for [AlC_8_H_4_O_5_]·4.87(D_2_O) returns to space group *I*4_1_22, consistent with an increased ordering upon the complete formation of encapsulated water clusters. This reflects the compact packing of water molecules driven by hydrogen bonding between guest molecules, with relatively weak host–guest interactions, as evidenced by the slightly longer hydrogen bonding distance between water and the bridging hydroxyl (H_hydroxyl_···O_D2O_ = 2.32 Å) in [AlC_8_H_4_O_5_]·4.87(D_2_O) compared with [AlC_8_H_4_O_5_]·1.89(D_2_O) (H_hydroxyl_···O_D2O_ = 2.01 Å). Similar structural features were also observed for MFM-300(Cr) (Figure S14 and Table S2). For the partially-loaded structure [CrC_8_H_4_O_5_]·2.01(D_2_O), solved and refined from NPD data, a space group of *P*4_1_22 was established, but on saturation, the structure of [CrC_8_H_4_O_5_]·3.67(D_2_O) showed a space group of *I*4_1_22. Thus, the results presented above provide valuable insights into the water adsorption profile and water-induced structural deformation of MOF materials.

**Figure 4:**
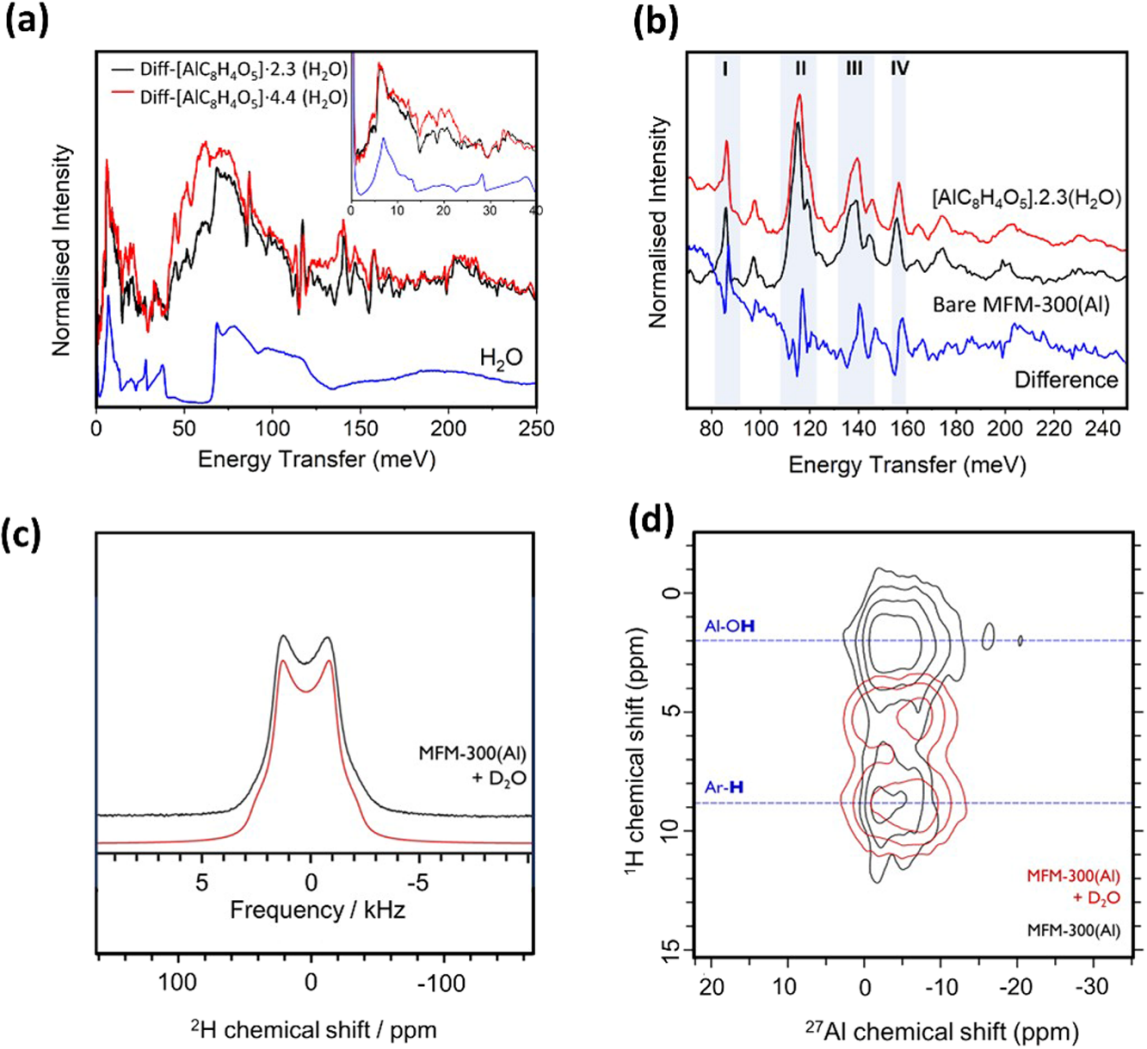
(a) INS spectra of ice, and the difference spectra of [AlC_8_H_4_O_5_]·2.3(H_2_O) and [AlC_8_H_4_O_5_]·4.4(H_2_O). (b) Comparison of the INS spectra of the framework region for the bare MOF and for [AlC_8_H_4_O_5_]·2.3(H_2_O). (c) Static ambient temperature ^2^H NMR spectrum of MFM-300(Al) saturated with D_2_O (black) and corresponding fit (red). (d) ^1^H–^27^Al 2D HETCOR NMR spectra of D_2_O-saturated (red) and pristine (black) MFM-300(Al), recorded with a MAS frequency of 12 kHz under ambient conditions.

INS spectra of activated MFM-300(Al) and H_2_O-loaded MFM-300(Al) were collected, and the difference spectra were obtained by subtracting the MOF background from the spectrum of H_2_O-loaded MOF (Figure 4). The features at lower energy (<80 meV) in the difference spectrum originate mainly from vibrational modes of the adsorbed water, and those at higher energy (80–160 meV) reflect changes in the local framework upon adsorption of water. Compared with the INS spectrum of ice, both the transitional (<40 meV) and librational (68–130 meV) modes shift toward lower energy for adsorbed water in MFM-300(Al). This is because adsorbed water molecules in the pore interact mainly with the framework (*via* hydroxyls) and adjacent water molecules (O···O distances: 2.43–3.88 Å), and these interactions are weaker compared with the 3-dimensional (3D) hydrogen bonding network within ice, where one water molecule forms 4 hydrogen bonds with adjacent water molecules (O···O distance: 2.76 Å).^49^ For the MOF region, four distinct features were observed in the difference spectrum, and these originate from broadening of the corresponding peaks in the spectrum of bare-MFM-300(Al). The observation that these peaks showed no change in energy but only broadened with marginal changes in their intensities is consistent with the adsorbed water molecules interacting only weakly with the host framework. The assignment of these features was enabled by DFT simulations of the INS spectra of bare and H_2_O-loaded MFM-300(Al), and good agreement was established between the simulated and experimental spectra (Figure S21) with the following assignments: Peak I: H_–OH_ rocking out of the Al–O–Al plane; Peak II: H_C6-ring_ wagging out of the C_6_ plane; Peak III: H_–OH_ rocking within the Al–O–Al plane; Peak IV: H_C6-ring_ wagging within the C_6_ plane. Upon loading of water, the correlated vibrations of the framework hydrogen atoms are disturbed by interactions with water molecules, and this results in broadening of the peaks. Overall, this is in excellent agreement with the structural analyses determined from the NPD data.

Previous studies have shown the power of ssNMR spectroscopy to investigate host–guest interactions, framework modifications upon guest incorporation, and guest dynamics in MOFs.^50^ Notably, water adsorption can be studied by ^2^H NMR spectroscopy when loading the MOF with D_2_O. Open metal sites bind strongly to water molecules, restricting their motion, and a broad Pake pattern (∼150 kHz) is observed.^51^ The dynamics of D_2_O molecules within the pores, especially with increased temperature, lead to an averaging of this Pake pattern, with a Lorentzian line shape resulting from fast isotropic motion.^51^ The ^2^H static NMR spectrum of MFM-300(Al) saturated with D_2_O at ambient temperature displays a motionally averaged Pake pattern with fwhm of 3.3 kHz (Figure 4c). However, the line shape is not Lorentzian, which indicates that the motion is not fully isotropic. The water likely undergoes rapid (>10^8^ s^–1^) rotation around its *C*_*2*_ axis as well as 3D hopping between adsorption sites and along the channels.^51^ Comparison of the ^1^H–^27^Al 2D magic angle spinning (MAS) heteronuclear dipolar correlation spectra of D_2_O-saturated MFM-300(Al) with pristine MFM-300(Al) shows that the water is hydrogen bonded to framework hydroxyls (Figure 4d). This is evidenced by the shifting of the correlation peak associated with the hydroxyls to a higher ^1^H chemical shift upon water loading. Notably, the line shape of the signal in the ^27^Al MAS NMR spectrum does not change substantially upon D_2_O adsorption, indicating that MFM-300(Al), unlike MIL-160,^11^ does not undergo large local structural changes upon water adsorption. Thus, the ssNMR analysis confirms that the binding of water on MFM-300(Al) is associated with the hydroxyls. This is weaker when compared to that of MOFs incorporating open metal sites. The pore size and channels enable specific adsorption and also facile regeneration with little structural change upon adsorption, demonstrating MFM-300(M) as a highly robust sorbent for water.

## Conclusions

Decarbonisation of cooling and heating by the recovery and utilization of waste heat critically relies on the development of sorbent materials. The materials MFM-300(M) (M = Al, Fe, Cr, V) are sorbents for water with desirable features for applications in adsorption-driven cooling. Both high working capacity and coefficient of performance (>0.8) have been achieved at an ultralow regeneration temperature (62 °C), thus establishing this series of MOFs as among the top-performing sorbents for this application. We further established structure–function relationships by conducting *in situ* NPD, INS/DFT, and ssNMR investigations of the MFM-300/water systems. Precise determination of the location of water molecules, including the positions of all deuterium centers, at different stages of adsorption provided a thorough view of the process of pore-filling, which unambiguously rationalized the observed S-shaped isotherms. These MFM-300(M) materials are also highly stable, can be readily manufactured, and have potential as advanced water sorbents to promote the development of adsorption-driven heat transfer technologies in achieving global Net-Zero targets.

## Supporting Information Available

Additional experimental details, enthalpy of adsorption data, adsorption isotherms, stability tests, neutron diffraction data, views of crystal structures, INS data, and ssNMR data (PDF)
