# Supplementary material for: MFM-300 as High-Performance Sorbents for Water-Adsorption-Driven Cooling
Source: J Am Chem Soc. 2025 Apr 8;147(15):12481–90. doi: 10.1021/jacs.4c16752 (PMC12006999; doi:10.1021/jacs.4c16752)
Supplement: Supplementary file 1 — ja4c16752_si_001.pdf [file ja4c16752_si_001.pdf]

Supplementary Information

MFM-300 as high-performance sorbents for water-adsorption-driven cooling

Xue Han[1,2*], Yinlin Chen[2], Jiangnan Li[2,3], Wanpeng Lu[2], Wenyuan Huang[2,3], Yuanjun Wang,[1] Guixiang Wang[1], Ivan da Silva[4], Yongqiang Cheng[5], Luke L. Daemen[5], Pascal Manuel[4], Anibal J. Ramirez-Cuesta[5], Daniel Lee[6], Sihai Yang[2,3*] and Martin Schröder[2*]

[1]College of Chemistry, Beijing Normal University, Beijing 100091, China

[2]Department of Chemistry, University of Manchester, Manchester, M13 9PL, UK

[3]College of Chemistry and Molecular Engineering, Beijing National Laboratory for Molecular Sciences, Peking University, Beijing, 100871, China

[4]ISIS Facility, Rutherford Appleton Laboratory, Chilton, OX11 0QX, UK

[5]Neutron Scattering Division, Neutron Sciences Directorate, Oak Ridge National Laboratory, Oak Ridge, TN 37831, USA

[6]Department of Chemical Engineering, University of Manchester, Manchester, M13 9PL, UK

Contents:

1. Materials and synthesis
2. Calculation of isosteric enthalpy of adsorption (ΔH_{ads})
3. Additional structural information on MFM-300(Al)
4. Evaluation of the stability of the materials
5. Assessment of performance of the materials for adsorption-driven cooling
6. Neutron powder diffraction
7. Inelastic neutron scattering
8. Solid state nuclear magnetic resonance spectroscopy
9. Attenuated total reflectance FT infrared spectroscopy
10. SEM images of MFM-300(M) (M = Al, Fe, Cr, V).

1. Materials and synthesis

Aluminium nitrate nonahydrate [$Al(NO_3)_3.9H_2O$, >98%, Sigma-Aldrich], iron chloride hexahydrate ($FeCl_3·6H_2O$, >97%, Sigma-Aldrich), chromium chloride hexahydrate ($CrCl_3·6H_2O$, > 98%, Sigma-Aldrich), vanadium chloride (VCl_3, 97%, Sigma-Aldrich), hydrochloric acid (HCl, 37%, Fisher Scientific), nitric acid (HNO_3, 70%, Fisher Scientific), dimethylformamide (DMF, > 99%, Fisher Scientific), acetone (> 99%, Sigma) were used as purchased. Biphenyl-3,3′,5,5′-tetracarboxylic acid (H_4BPTC) was synthesised with literature method.[1]

MFM-300(Al): Synthesis of MFM-300(Al) was carried out using a modification of the previously reported method.[2] H_4BPTC (0.6 g, 1.8 mmol), $Al(NO_3)_3.9H_2O$ (3.4 g, 9.1 mmol) and piperazine (1.0 g, 12.6 mmol) were mixed and dispersed in water (100 mL), and HNO_3 (2.8 M, 20 mL) added to the resulting white slurry. The slurry was transferred to a 300 mL Teflon-lined autoclave, giving a liquid to atmosphere ratio of 0.67 within the reactor, which was sealed and heated to 483 K for 3 days. The reactor was allowed to cool to room temperature, and the resultant white microcrystalline product MFM-300(Al) was separated by filtration and washed with water. Water was exchanged with acetone three times a day for 3 days before the activation of the sample under dynamic vacuum at 393 K.

MFM-300(Fe): Synthesis of MFM-300(Fe) was carried out using a modification of the previously reported method.[3] H_4BPTC (244 mg, 0.74 mmol), $FeCl_3·6H_2O$ (800 mg, 2.96 mmol) were dispersed in a mixture of DMF (20 mL) and HCl (36.5 wt%, 0.75 mL) in a 50 mL glass round-bottom flask under ambient pressure. The liquid to atmosphere ratio of the reaction system was 0.4. The reaction mixture was heated at 120 °C for 72 h, and the resultant yellow powder precipitate of MFM-300(Fe) was separated by centrifugation and washed with DMF and acetone and dried in air.

MFM-300(Cr): Synthesis of MFM-300(Cr) was carried out using the previously reported method.[4] H_4BPTC (0.70 g, 2.1 mmol), $CrCl_3·6H_2O$ (2.83 g, 10.6 mmol) and deionised water 100 mL were mixed, and HCl (1%, 15 mL) added. The mixture was transferred into a 250 mL Teflon-lined autoclave, which was sealed and heated to 483 K for 3 days. The liquid to atmosphere ratio of the reaction system was 0.85. After the reaction, the reactor was allowed to cool to room temperature, and the resultant blue microcrystalline product of MFM-300(Cr) was separated by centrifugation and stored under acetone.

MFM-300(V): Synthesis of MFM-300(V^{III}) was carried out using previously reported method.[4] H_4BPTC (1.12 g, 3.4 mmol), VCl_3 (3.20 g, 20.3 mmol) and 80 mL deionised water were mixed, and HCl (1%, 2 mL) added. N_2 was passed through the mixture for 5 mins, which was transferred into a 200 mL Teflon-lined autoclave, sealed and heated to 483 K for 3 days. The liquid to atmosphere ratio of the reaction system was 0.69. The reaction was allowed to cool to room temperature, and the resultant green microcrystalline product of MFM-300(V^{III}) was separated by centrifugation and washed by acetone. The product was stored under acetone.

2. Calculation of isosteric enthalpy of adsorption (ΔH_{ads})

The isosteric enthalpy of adsorption (ΔH_{ads}) as a function of loading of water vapor (n) was calculated from three isotherms (10 °C, 20 °C and 30 °C) using the Clausius-Clapeyron approach.[5] This is by far the most common way to derive ΔH_{ads} of gas adsorption with MOF materials, and has been applied to many gases such as H_2, CO_2, CH_4, and H_2O. This method requires the measurement of at least two adsorption isotherms at close but different temperatures, usually the difference being 10-20 °C.

$$\ln (p)_n = \frac{\Delta H_n}{RT} - \frac{\Delta S_n}{R}$$

A plot of $\ln(p)$ versus $1/T$ at constant loading allows the isosteric enthalpy of adsorption (ΔH_{ads}, n) to be determined. Figure S1-S8 show the fitted plots of $\ln(p)$ versus 1/T and the calculated values for ΔH_{ads} for each MOF at specific loadings of water.

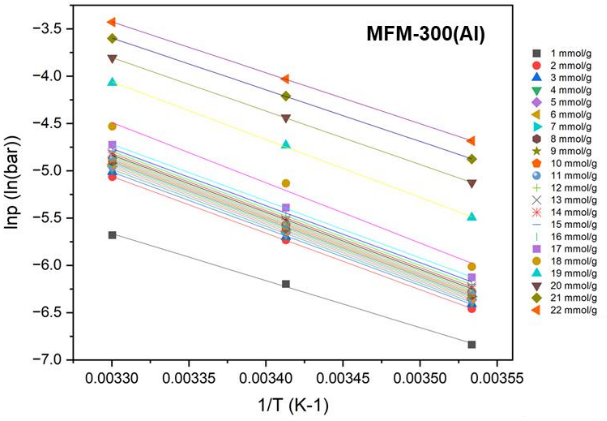

Loading (mmol/g)	Intercept		Slope		Statistics
	Value	Standard Error	Value	Standard Error	Adj. R-Square
1	10.74	0.72	-4971.00	212.02	0.9964
2	14.68	0.06	-5982.73	18.26	1.0000
3	14.75	0.20	-5988.85	59.86	0.9998
4	14.91	0.28	-6028.05	83.03	0.9996
5	14.98	0.34	-6043.04	98.98	0.9995
6	15.03	0.37	-6051.76	108.80	0.9994
7	15.06	0.39	-6056.99	115.07	0.9993
8	15.09	0.41	-6059.98	118.96	0.9992
9	15.11	0.41	-6061.36	121.02	0.9992
10	15.12	0.42	-6061.44	121.47	0.9992
11	15.13	0.41	-6060.36	120.27	0.9992
12	15.14	0.40	-6058.11	117.13	0.9993
13	15.15	0.38	-6054.55	111.34	0.9993
14	15.15	0.35	-6049.36	101.38	0.9994
15	15.14	0.29	-6041.92	83.63	0.9996
16	15.14	0.16	-6031.12	47.37	0.9999
17	15.14	0.20	-6016.49	58.63	0.9998
18	16.55	1.92	-6376.14	561.10	0.9846
19	16.12	0.44	-6113.66	130.15	0.9991
20	14.86	0.13	-5656.61	37.08	0.9999
21	14.45	0.09	-5469.65	27.48	1.0000
22	14.35	0.10	-5385.69	28.29	0.9999

Figure S1. Plots of ln(p) versus 1/T at different water loadings in MFM-300(Al) and associated data.

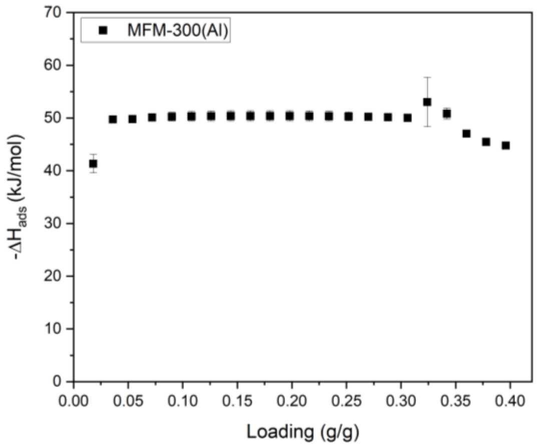

loading (mmol/g)	loading (g/g)	$-\Delta H_{ads}$ (kJ/mol)	$-\Delta H_{ads}$ Error (kJ/mol)
1	0.02	41.33	1.76
2	0.04	49.74	0.15
3	0.05	49.79	0.50
4	0.07	50.12	0.69
5	0.09	50.24	0.82
6	0.11	50.31	0.90
7	0.13	50.36	0.96
8	0.14	50.38	0.99
9	0.16	50.39	1.01
10	0.18	50.39	1.01
11	0.20	50.39	1.00
12	0.22	50.37	0.97
13	0.23	50.34	0.93
14	0.25	50.29	0.84
15	0.27	50.23	0.70
16	0.29	50.14	0.39
17	0.31	50.02	0.49
18	0.32	53.01	4.66
19	0.34	50.83	1.08
20	0.36	47.03	0.31
21	0.38	45.47	0.23
22	0.40	44.78	0.24

Figure S2. Isosteric enthalpy for water adsorption in MFM-300(Al) at different water loadings and associated data.

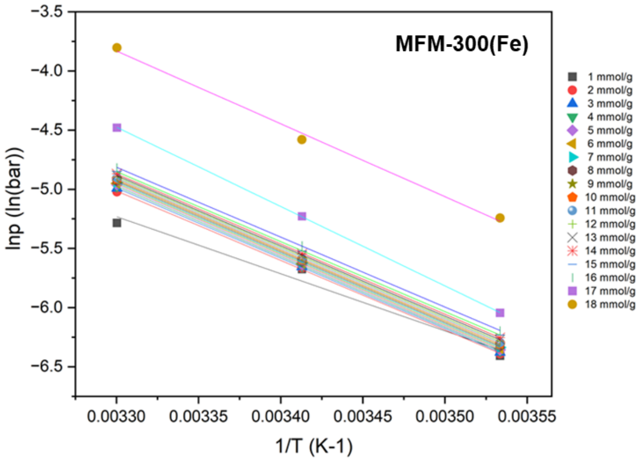

	Intercept		Slope		Statistics
Loading (mmol/g)	Value	Standard Error	Value	Standard Error	Adj. R-Square
1	10.71	2.56	-4829.83	749.68	0.9530
2	14.38	0.31	-5876.57	91.47	0.9995
3	14.63	0.11	-5943.39	32.46	0.9999
4	14.73	0.01	-5969.33	2.42	1.0000
5	14.78	0.06	-5980.87	16.20	1.0000
6	14.80	0.10	-5985.45	28.76	1.0000
7	14.81	0.13	-5986.01	37.59	0.9999
8	14.82	0.15	-5983.93	43.81	0.9999
9	14.81	0.16	-5979.87	48.05	0.9999
10	14.80	0.17	-5974.16	50.60	0.9999
11	14.79	0.18	-5966.86	51.48	0.9999
12	14.77	0.17	-5957.89	50.48	0.9999
13	14.74	0.16	-5946.97	46.87	0.9999
14	14.71	0.13	-5933.62	38.82	0.9999
15	14.68	0.07	-5917.64	20.66	1.0000
16	14.68	0.12	-5907.47	35.42	0.9999
17	17.67	0.12	-6709.86	34.36	1.0000
18	16.50	1.36	-6159.63	399.16	0.9916

Figure S3. Plots of ln(p) versus 1/T at different water loadings in MFM-300(Fe) and associated data.

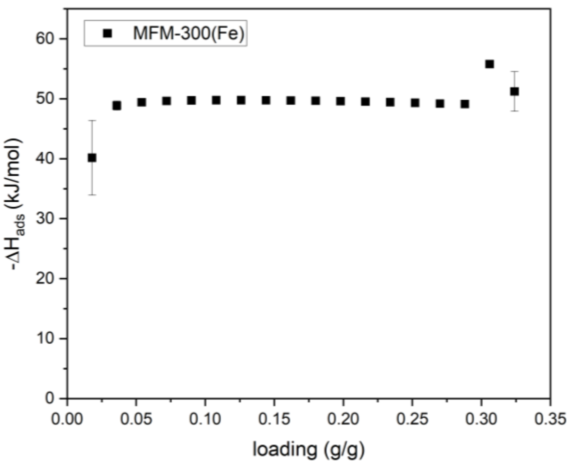

loading (mmol/g)	loading (g/g)	$-\Delta H_{ads}$ (kJ/mol)	$-\Delta H_{ads}$ Error (kJ/mol)
1	0.02	40.16	6.23
2	0.04	48.86	0.76
3	0.05	49.41	0.27
4	0.07	49.63	0.02
5	0.09	49.72	0.13
6	0.11	49.76	0.24
7	0.13	49.77	0.31
8	0.14	49.75	0.36
9	0.16	49.72	0.40
10	0.18	49.67	0.42
11	0.20	49.61	0.43
12	0.22	49.53	0.42
13	0.23	49.44	0.39
14	0.25	49.33	0.32
15	0.27	49.20	0.17
16	0.29	49.11	0.29
17	0.31	55.79	0.29
18	0.32	51.21	3.32

Figure S4. Isosteric enthalpy for water adsorption in MFM-300(Fe) at different water loadings and associated data.

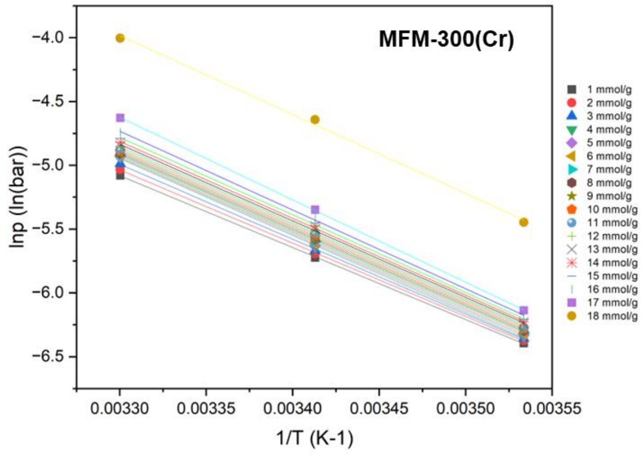

	Intercept		Slope		Statistics
Loading (mmol/g)	Value	Standard Error	Value	Standard Error	Adj. R-Square
1	13.52	0.15	-5637.62	45.14	0.9999
2	13.95	0.20	-5752.6	58.30	0.9998
3	14.40	0.31	-5876.9	91.84	0.9995
4	14.94	0.50	-6024.96	147.30	0.9988
5	14.87	0.35	-6001.33	103.50	0.9994
6	14.82	0.23	-5983.97	66.22	0.9998
7	14.79	0.12	-5971.77	33.73	0.9999
8	14.77	0.02	-5964.14	4.92	1.0000
9	14.77	0.07	-5960.84	20.97	1.0000
10	14.79	0.15	-5961.98	44.43	0.9999
11	14.82	0.22	-5968.02	65.79	0.9998
12	14.88	0.29	-5979.98	85.17	0.9996
13	14.96	0.35	-5999.75	102.48	0.9994
14	15.09	0.40	-6031.1	117.07	0.9993
15	15.29	0.43	-6082.58	126.86	0.9991
16	15.66	0.42	-6179.58	123.64	0.9992
17	16.73	0.15	-6469.3	43.45	0.9999
18	16.45	1.00	-6192.11	292.47	0.9956

Figure S5. Plots of ln(p) versus 1/T at different water loadings in MFM-300(Cr) and associated data.

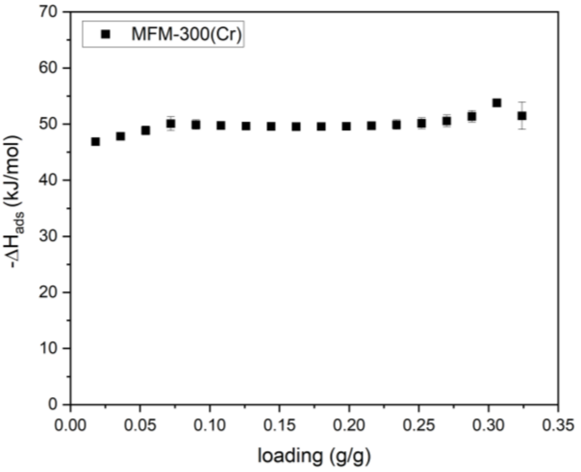

loading (mmol/g)	loading (g/g)	$-\Delta H_{ads}$ (kJ/mol)	$-\Delta H_{ads}$ Error (kJ/mol)
1	0.02	46.87	0.38
2	0.04	47.83	0.48
3	0.05	48.86	0.76
4	0.07	50.09	1.22
5	0.09	49.90	0.86
6	0.11	49.75	0.55
7	0.13	49.65	0.28
8	0.14	49.59	0.04
9	0.16	49.56	0.17
10	0.18	49.57	0.37
11	0.20	49.62	0.55
12	0.22	49.72	0.71
13	0.23	49.88	0.85
14	0.25	50.14	0.97
15	0.27	50.57	1.05
16	0.29	51.38	1.03
17	0.31	53.79	0.36
18	0.32	51.48	2.43

Figure S6. Isosteric enthalpy for water adsorption in MFM-300(Cr) at different water loadings and associated data.

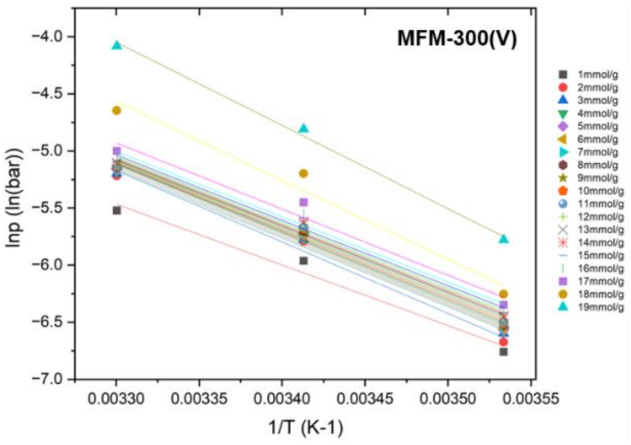

	Intercept		Slope		Statistics
Loading (mmol/g)	Value	Standard Error	Value	Standard Error	Adj. R-Square
1	12.08	2.65	-5316.77	776.07	0.9583
2	12.08	2.65	-5316.77	776.07	0.9583
3	15.49	2.17	-6260.3	634.99	0.9796
4	14.68	1.72	-6013.31	502.74	0.9861
5	14.75	1.71	-6029.37	500.31	0.9863
6	14.83	1.68	-6048.28	491.14	0.9869
7	14.77	1.72	-6025.46	504.27	0.9861
8	14.65	1.80	-5986.56	525.38	0.9847
9	14.53	1.86	-5949.88	544.12	0.9834
10	14.42	1.92	-5914.86	561.34	0.9822
11	14.31	1.97	-5881.03	577.80	0.9809
12	14.21	2.03	-5848.01	594.24	0.9796
13	14.11	2.09	-5815.52	611.66	0.9781
14	14.02	2.16	-5783.46	631.64	0.9764
15	13.93	2.25	-5752.22	657.23	0.9742
16	13.85	2.38	-5723.9	695.86	0.9709
17	13.83	2.64	-5708.84	771.60	0.9641
18	14.20	3.39	-5795.84	992.88	0.9430
19	18.26	3.81	-6916.67	1115.77	0.9493
20	19.99	1.55	-7285.37	454.01	0.9923

Figure S7. Plots of ln(p) versus 1/T at different water loadings in MFM-300(V) and associated data.

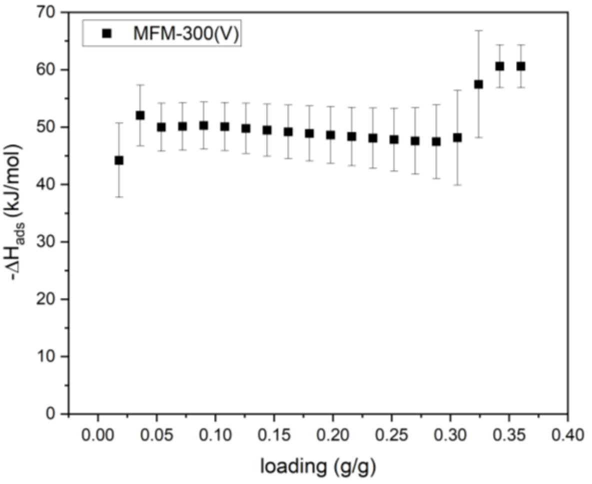

Loading (mmol/g)	loading (g/g)	$-\Delta H_{ads}$ (kJ/mol)	$-\Delta H_{ads}$ Error (kJ/mol)
1	0.02	44.20	6.45
2	0.04	52.05	5.28
3	0.05	49.99	4.18
4	0.07	50.13	4.16
5	0.09	50.29	4.08
6	0.11	50.10	4.19
7	0.13	49.77	4.37
8	0.14	49.47	4.52
9	0.16	49.18	4.67
10	0.18	48.90	4.80
11	0.20	48.62	4.94
12	0.22	48.35	5.08
13	0.23	48.09	5.25
14	0.25	47.83	5.46
15	0.27	47.60	5.78
16	0.29	47.47	6.41
17	0.31	48.16	8.27
18	0.32	57.46	9.31

Figure S8. Isosteric enthalpy for water adsorption in MFM-300(V) at different water loadings and associated data.

3. Additional structural information on MFM-300(Al)

MFM-300(Al) shows structural similarities to several reported Al-based MOFs, which are composed of μ-OH and carboxylate bridged {AlO_6} octahedra shared vertices through the μ-OH bridge. The polymorphism of the AlO_6 octahedra has been found to have a marked effect on the hydrophilicity of the material. For example, MIP-211, composed of *cis*-connected corner-sharing AlO_6 octahedra and t,t'-muconate, has a much higher hydrophilicity than MIL-53-muc, composed of *trans*-connected corner-sharing AlO_6 octahedra and t,t'-muconate.[6] MFM-300(Al) has *cis*-connected corner-sharing AlO_6 octahedra as found in CAU-10, MIL-160, KMF-1 and MIP-211, and the measured inflection point of the water adsorption isotherm for MFM-300(Al) ($p/p0 = 0.2$) is close to that of MIP-211, demonstrating a relatively high hydrophilicity for MFM-300(Al).

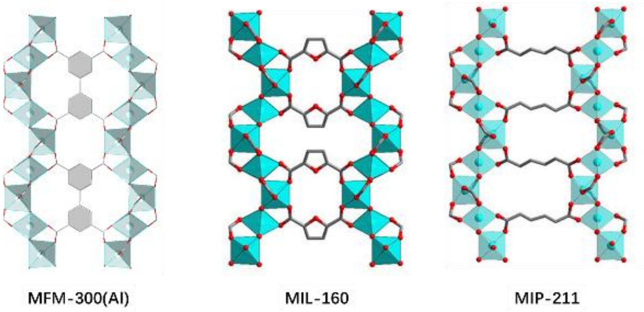

Figure S9. Comparison the corner-sharing AlO_6 octahedra of MFM-300(Al), MIL-160 and MIP-211.

4. Evaluation of the stability of the materials

PXRD of the samples (as-synthesised, sample that was immersed in water at room temperature for one month, sample that was immersed in boiling water for five days, sample that was directly activated from water, and sample after 15 cycles of adsorption/desorption of water) were recorded on a Philips X'pert X-ray diffractometer (40 kV and 30 mA) using Cu-Kα radiation ($\lambda = 1.5406$ Å). The data were collected at room temperature over a 2θ range of 5–50° with a scan speed of 4° min^{-1}. MFM-300(M) (M = Al, Fe, Cr) retained their crystallinity after one month in water at room temperature, in boiling water for five days, and over 15 cycles of adsorption/desorption of water, but showed decreased crystallinity after direct activation from water at 110 °C under dynamic vacuum. For MFM-300(V), crystallinity reduced after being retained in boiling water for five days, and after 15 cycles of adsorption/desorption of water and also by direct activation from water.

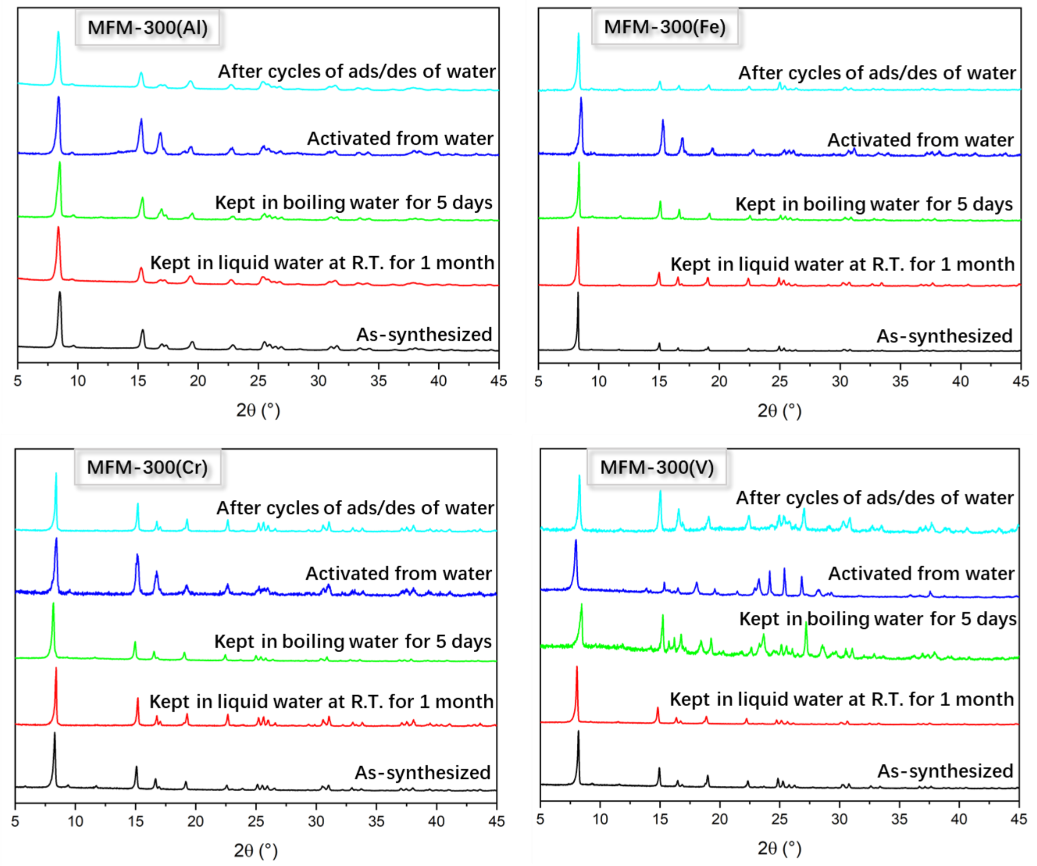

Figure S10. PXRD patterns of MFM-300(M) (M = Al, Fe, Cr, V) after different sample treatments.

Adsorption isotherms for CO_2 in these materials at 273 K were also measured with as-synthesised samples activated from acetone and from water. All samples were activated at 180 °C under dynamic vacuum for 24 h before measurement of isotherms. As shown in Figure S11, samples that are activated from water showed lower CO_2 uptake than those of activated from acetone. Adsorption isotherms for water in MFM-300(M) (M = Al, Fe, Cr, V) were also measured on samples that had been kept under boiling water for 5 days, and these were compared with the isotherms of the as-synthesised, activated samples. MFM-300(M) (M = Al, Fe, Cr) showed the same features and very similar water uptakes between the fresh samples and those treated under boiling water. MFM-300(V) exhibited a later step in the isotherm, with greater hysteresis and lower uptake for the sample treated under boiling water. Combined with the PXRD data shown in Figure S10, these results demonstrate that direct activation from water caused some collapse of all frameworks, particularly to MFM-300(Al), whereas boiling water treatment has the greatest effect on MFM-300(V). It is thus necessary to exchange water with acetone multiple times prior to activation to ensure good crystallinity and high surface areas for the activated material.

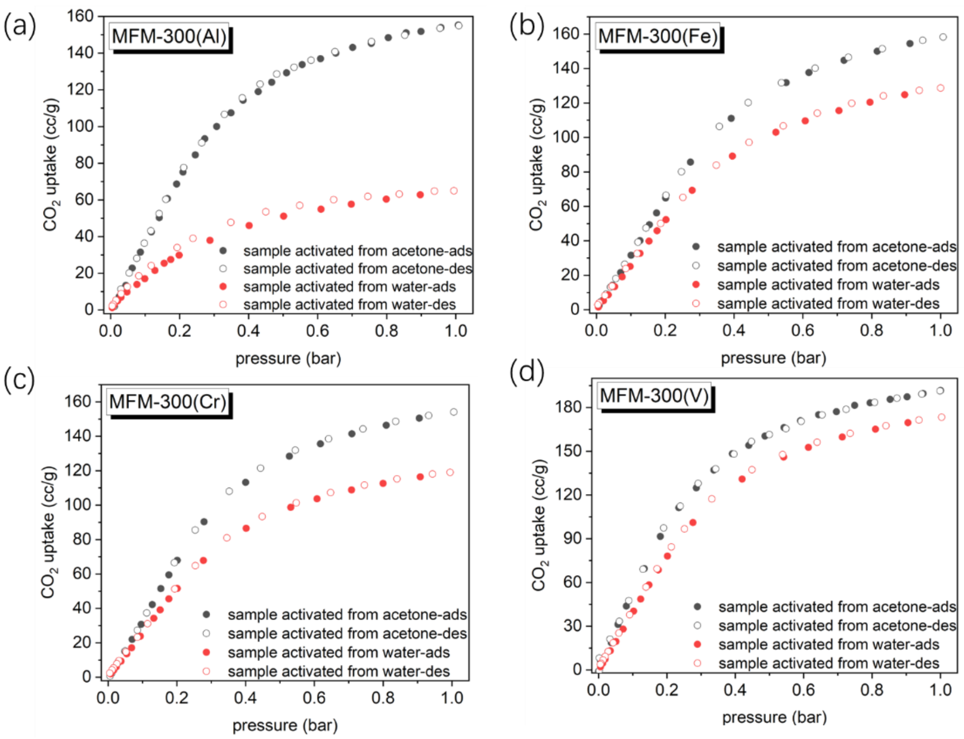

Figure S11. Adsorption isotherm of CO_2 at 273 K in MFM-300(M) (M=Al, Fe, Cr, V) activated from acetone and water.

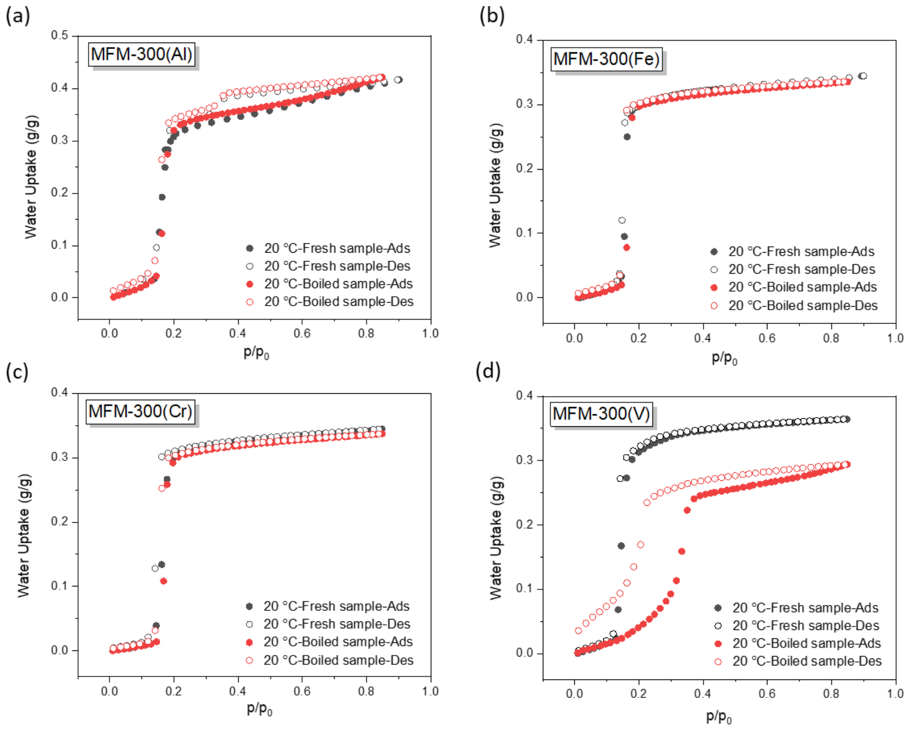

Figure **S12a**. Adsorption isotherms of water in MFM-300(M) (M = Al, Fe, Cr, V). Black: freshly synthesised samples; red: samples that had been treated under boiling water for 5 days.

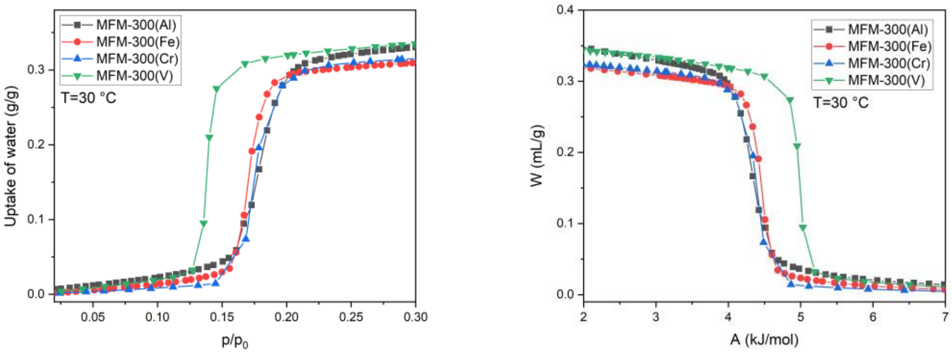

Figure **S12b**. Left: Comparison of adsorption isotherms of water for MFM-300(M) (M = Al, Fe, Cr, V) at 30°C; Right: Comparison of potential curves for water adsorption for MFM-300(M) (M = Al, Fe, Cr, V) at 30°C.

5. Assessment of performance of the materials for adsorption-driven cooling

(a) Characteristic curves

Due to limitations of instrumentation, adsorption isotherms were measured at 10, 20 and 30 °C in this work. This is lower than the temperatures used for desorption in adsorption-driven heat transfer, which is almost always above 60 °C. This means that the measured isotherms cannot be used directly in the models for the calculation of working capacities and coefficient of performances. To circumvent this, the concept of the characteristic curve was adopted, where the molar Gibbs free energy of adsorption with opposite sign is defined as adsorption potential (A)

$$A = RT \ln\left(\frac{P_0(T)}{P}\right)$$

A: adsorption potential

R: ideal gas constant

T: temperature at which the isotherm is measured

P_0: saturation vapor pressure of water at T

P: vapour pressure of water

The volume of the adsorbed water (W) that is derived from the adsorption isotherms is then plotted against A to give the characteristic curves (W-A curves). As seen in Figure S13, for MFM-300(M) (M = Al, Fe, Cr, V), the characteristic curves at three temperatures collapse onto one curve, thus allowing the determination of W at temperatures out of the range of measured isotherms from the characteristic curves. Additional detailed discussion on the validation of such characteristic curves for MOFs and other porous sorbents can be found in a recent reviews.[7,8]

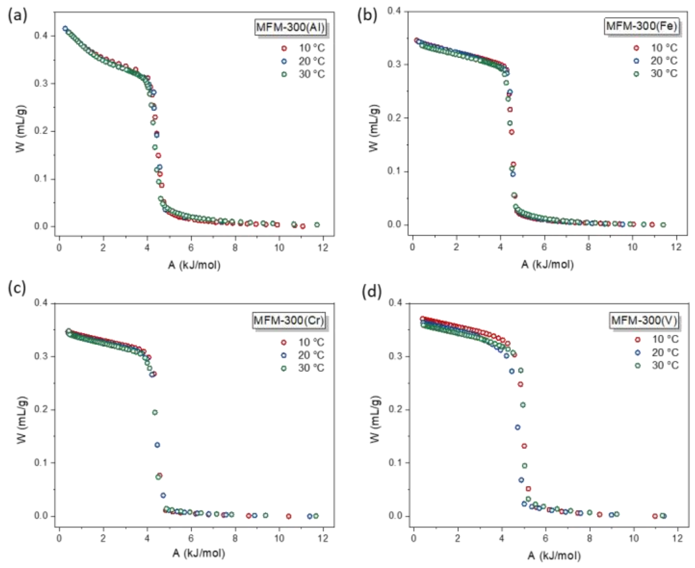

Figure S13. Characteristic curves for MFM-300(M) (M = Al, Fe, Cr, V) determined using adsorption isotherms at 283 K, 293 K and 303 K.

(b) Working capacity (ΔW)

ΔW is defined as the difference in working fluid between the maximum and minimum isosteres (line ①-② and line ③-④ in Figure 1). W_{max} is the amount of water that is adsorbed within MOF at point ①, and W_{min} is the uptake at point ③. With set T_{con} and T_{ev}, W_{max} is fixed and W_{min} depends on T_{des}.

$$W = \frac{q(p,T)}{\rho(T)}$$

W: the volume liquid adsorbed

q: the mass adsorbed

$\rho(T)$: density of liquid water

(c) Coefficient of performance

The coefficient of performance (COP) for cooling is defined as the ratio of evaporation enthalpy of the liquid phase and the required heat for regeneration. A full description of thermal dynamic models and associated detailed calculations are described in the literature,[7] noting that based on this definition, for a single adsorption bed, the maximum theoretical COP for cooling is 1.

$$\text{COP} = \frac{Q_{ev}}{Q_{regen}}$$

Table S1. Summary of the working capacity (ΔW) and COP of some recently reported MOF/water working pair for adsorption-driven cooling under two sets of temperature boundaries.

For refrigeration<br>T_{ev} = 5 °C, T_{con} = T_{ads} = 30 °C, and T_{des} =70 °C				For air-conditioning<br>T_{ev} = 10 °C, T_{con} = T_{ads} = 30 °C, and T_{des} =70 °C			
Sorbents	ΔW (mL/mL)	COP	Ref	Sorbents	ΔW (mL/mL)	COP	Ref
CAU-23	0.01	0.4	[9]	MIL-53(Cr)	0.07	0.41	[7]
MIL-160	0.12	0.62		MOF-801	0.16	0.57	
SAPO-34	0.13	0.62		AQSOA-Z02	0.18	0.66	
MIP-200	0.17	0.72		AQSOA-Z01	0.25	0.71	
MOF-303	0.26	0.72		Zn(BDC)(DABAO)$_{0.5}$	0.25	0.68	
CAU-10	0.3	0.75		Activated Carbon	0.29	0.55	
KMF-1	0.36	0.75		CAU-10-H	0.33	0.75	
Co-CUK-1	0.34	0.82		MOF-841	0.47	0.82	
KMF-2	0.34	0.75	[10]	MFM-300(Al)	0.31	0.82	This work
EMM-8	0.38	0.85	[11]	MFM-300(Fe)	0.29	0.82	This work
MFM-300(Al)	0.29	0.82	This work	MFM-300(Cr)	0.32	0.83	This work
MFM-300(Fe)	0.32	0.82	This work	MFM-300(V)	0.31	0.82	This work
MFM-300(Cr)	0.33	0.83	This work				
MFM-300(V)	0.33	0.82	This work				

6. Neutron powder diffraction

Structural determination of the binding position of D_2O within MFM-300(M) (M = Al, Cr) were conducted at WISH, a long wavelength powder and single crystal neutron diffractometer at the ISIS neutron and muon facility at Rutherford Appleton Laboratory (UK). Rietveld refinements of the NPD data for the bare MOF and for D_2O-loaded samples were performed using the TOPAS software package.[12] In this treatment the guest molecules were treated as rigid bodies, and the centres of mass, orientations, and occupancies of the adsorbate were refined first. This was followed by full profile Rietveld refinement including the positions of metal centers and linkers, together with their corresponding lattice parameters. This resulted in satisfactory R-factors. The final refinements on all the parameters including fractional coordinates, thermal parameters, occupancies for both host lattice and adsorbate molecule, and background/profile coefficients yielded very good agreement factors.

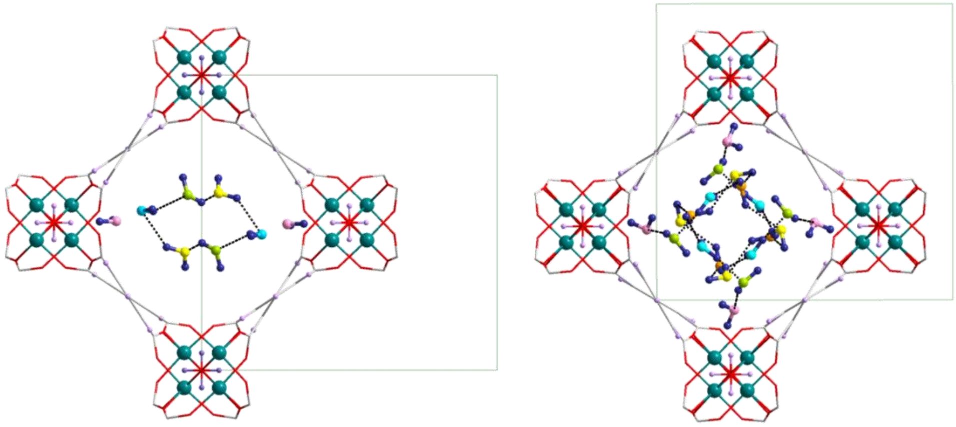

Figure S14. Views of structure of (a) $[CrC_8H_4O_5]\cdot 2.01(D_2O)$, and (b) $[CrC_8H_4O_5]\cdot 3.67(D_2O)$ derived from NPD data.

Table S2 Crystal Data and Details of the Structure Determination for D_2O-loaded MFM-300(M) (M = Al, Cr)

Moiety Formula	$C_8 H_4 D_{0.78} Al O_{5.39}$	$C_{16} H_8 D_{7.528} Al_2 O_{13.764}$	$C_8 H_4 D_{9.74} Al O_{9.87}$
Formula	$[AlC_8H_4O_5]\cdot 0.39(D_2O)$	$\{[AlC_8H_4O_5]\cdot 1.89(D_2O)\}_2$	$[AlC_8H_4O_5]\cdot 4.87(D_2O)$
Loading (D_2O:Al)	0.39	1.89	4.87
Formula Weight	214.94	489.54	304.62
Crystal System	Tetragonal	Tetragonal	Tetragonal
Space Group	$I4_122$	$P4_122$	$I4_122$
a, *b* (Å)	14.8199(14)	14.8160(12)	14.8350(7)
c (Å)	11.8231(12)	11.8046(10)	11.7730(5)
Volume (Å^3)	2596.7(6)	2591.3(5)	2591.0(3)
Z	8	4	8
ρ (calc) g/cm^3	1.0957	1.2555	1.5615
Radiation type	Neutron	Neutron	Neutron
Scan Method	Time of Flight	Time of Flight	Time of Flight
R_{exp}	0.338	0.324	0.308
R_{wp}	1.972	1.407	0.896
R_p	1.386	1.108	0.725
GoF	5.829	4.343	2.914
CCDC	2203274	2203275	2203276

Moiety Formula	$C_8 H_4 D_{4.02} Cr O_{7.01}$	$C_8 H_4 D_{7.33} Cr O_{8.67}$
Formula	$\{[CrC_8H_4O_5]\cdot 2.01(D_2O)\}_2$	$[CrC_8H_4O_5]\cdot 3.67(D_2O)$
Loading (D_2O:Cr)	2.01	3.67
Formula Weight	544.74	305.50
Crystal System	Tetragonal	Tetragonal
Space Group	$P4_122$	$I4_122$
a, *b* (Å)	14.9901(7)	15.0265(3)
c (Å)	11.9682(6)	11.9539(3)
Volume (Å^3)	2689.3(3)	2699.13(14)
Z	4	8
ρ (calc) g/cm^3	1.345	1.504
Radiation type	Neutron	Neutron
Scan Method	Time of Flight	Time of Flight
R_{exp}	0.350	0.303
R_{wp}	1.935	0.614
R_p	1.492	0.505
GoF	5.528	2.023
CCDC	2203277	2203278

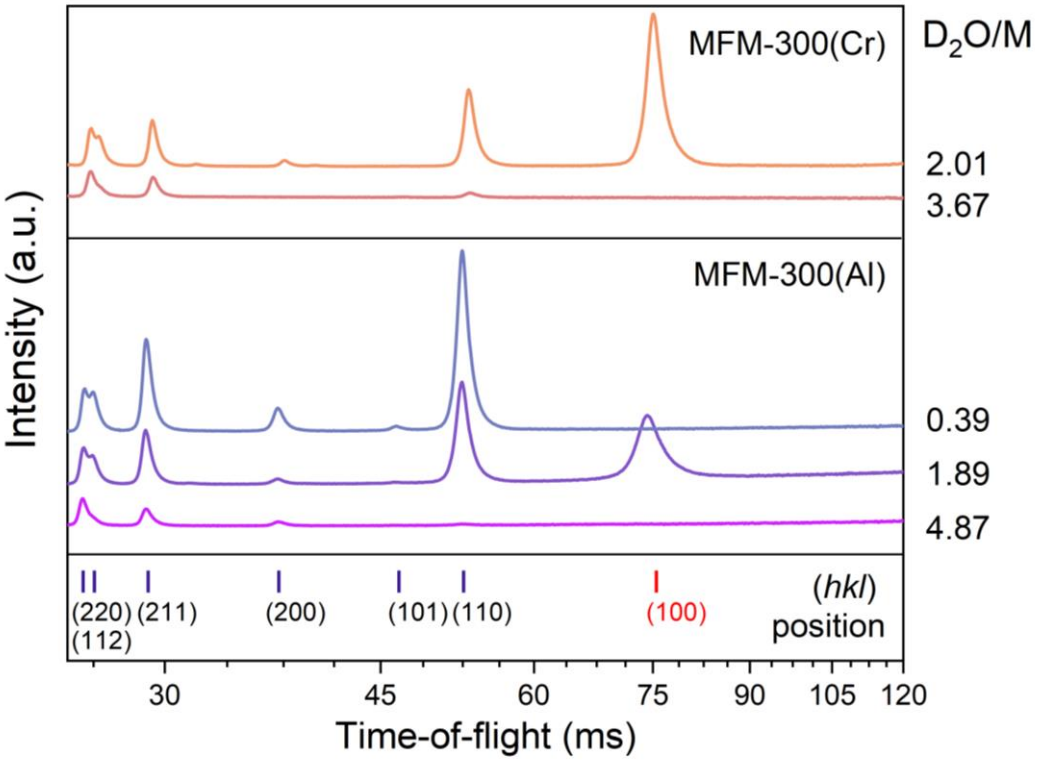

Figure S15. Neutron diffraction patterns of water-loaded MFM-300(Al) and MFM-300(Cr) on Bank 1. The amount of loading is marked on the right hand side. In water-loaded MFM-300(Al) (D_2O:Al = 1.89) and MFM-300(Cr) (D_2O:Cr = 2.01), an additional peak appears at ~75 ms, assigned to (100) thus confirming phase transition from $I4_122$ to $P4_122$.

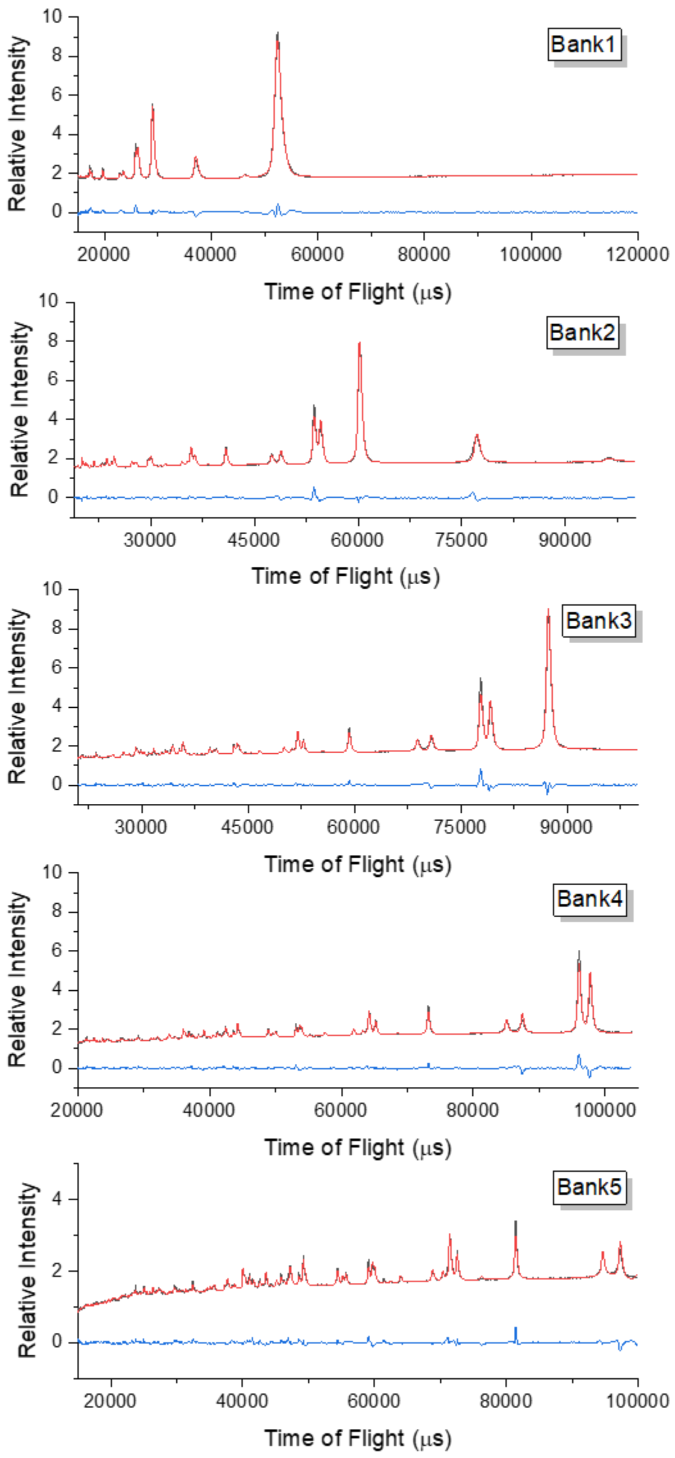

Figure S16. Neutron diffraction patterns and Rietveld refinement for $[AlC_8H_4O_5]\cdot 0.39(D_2O)$.

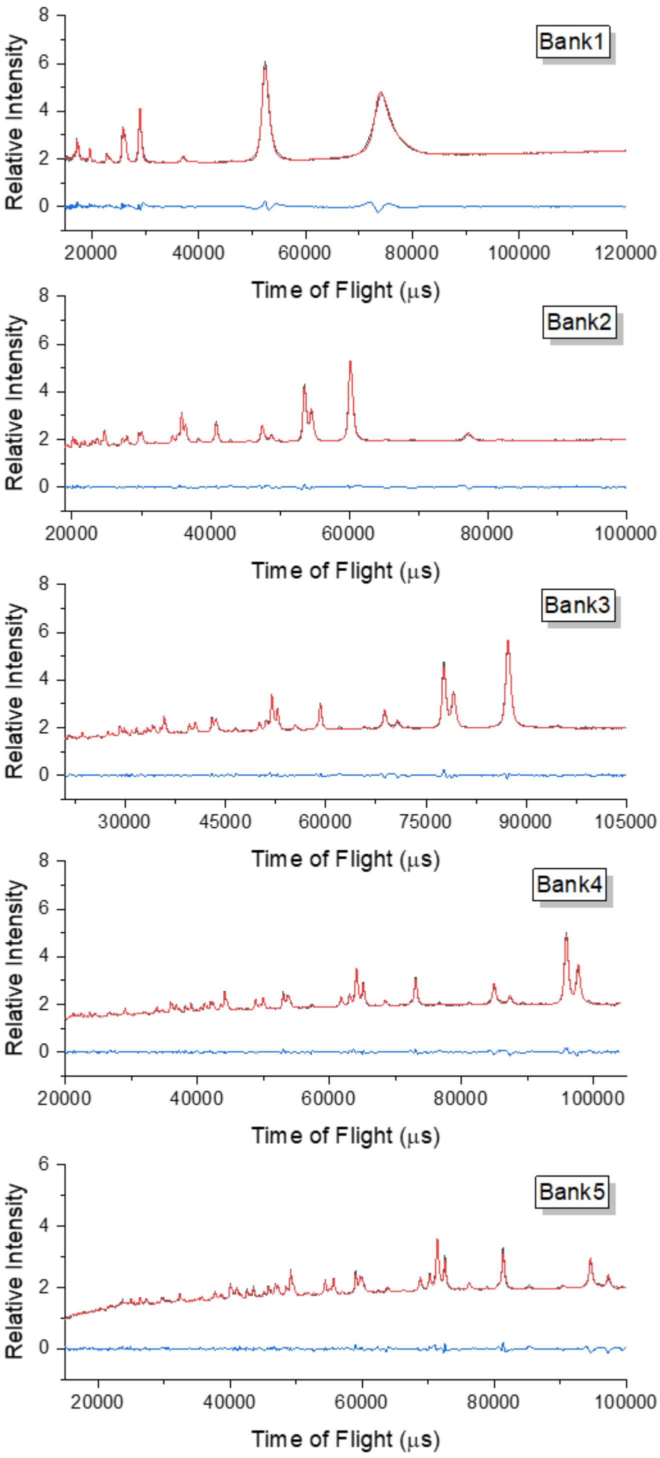

Figure S17. Neutron diffraction patterns and Rietveld refinement for [$AlC_8H_4O_5$]·1.89(D_2O).

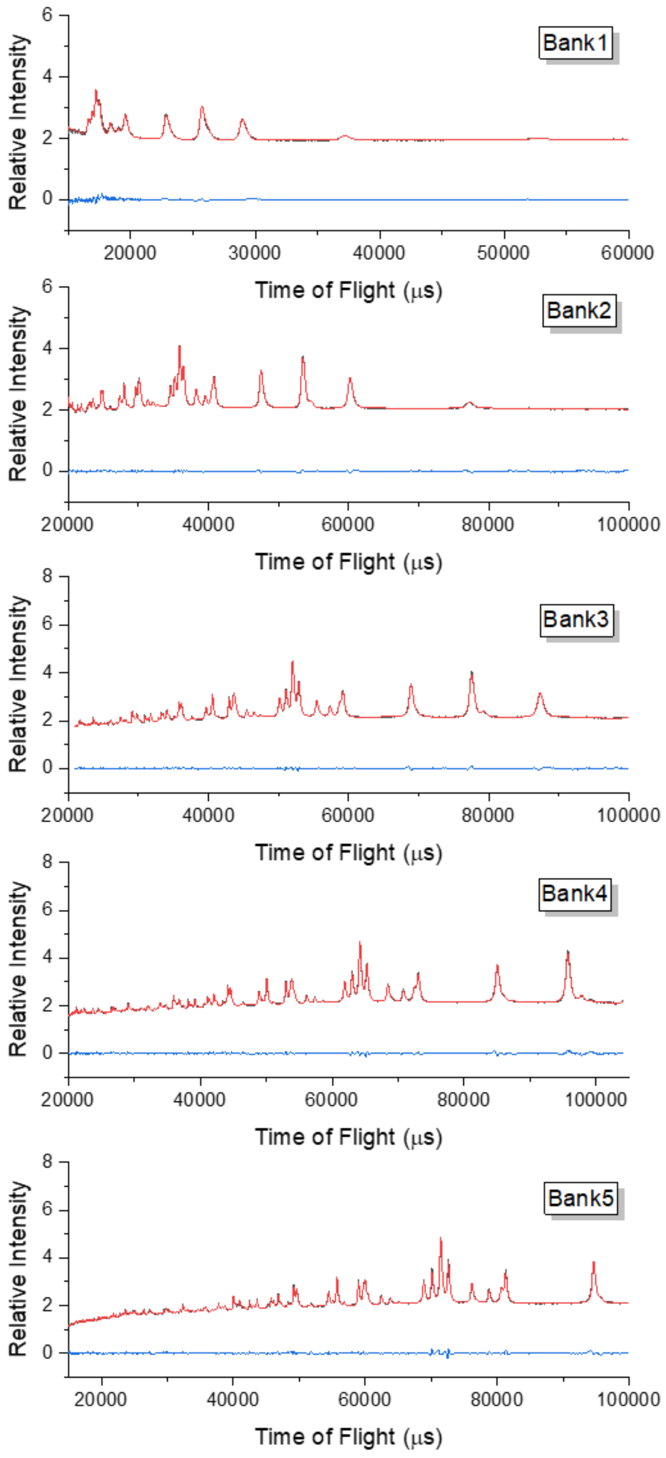

Figure S18. Neutron diffraction patterns and Rietveld refinement for [$AlC_8H_4O_5$]·4.87(D_2O).

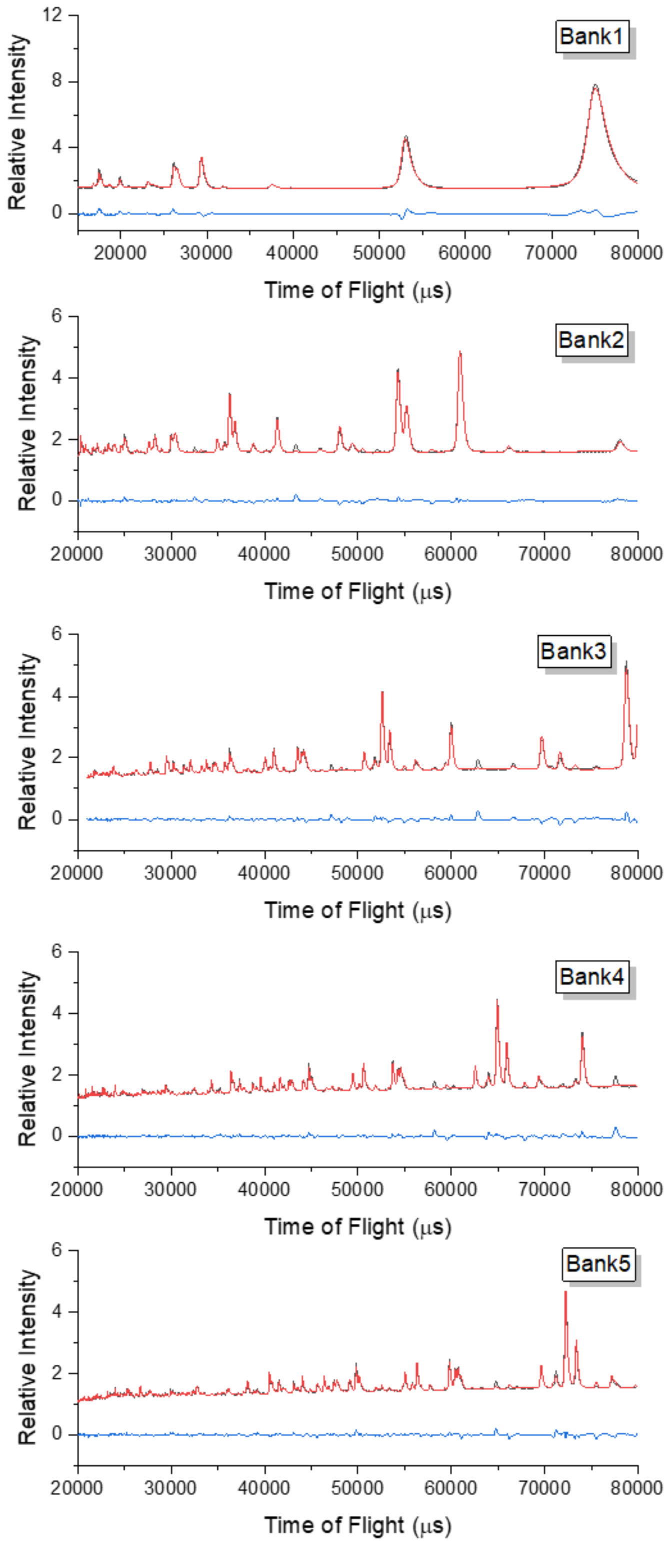

Figure S19. Neutron diffraction patterns and Rietveld refinement for $[CrC_8H_4O_5]\cdot 2.01(D_2O)$.

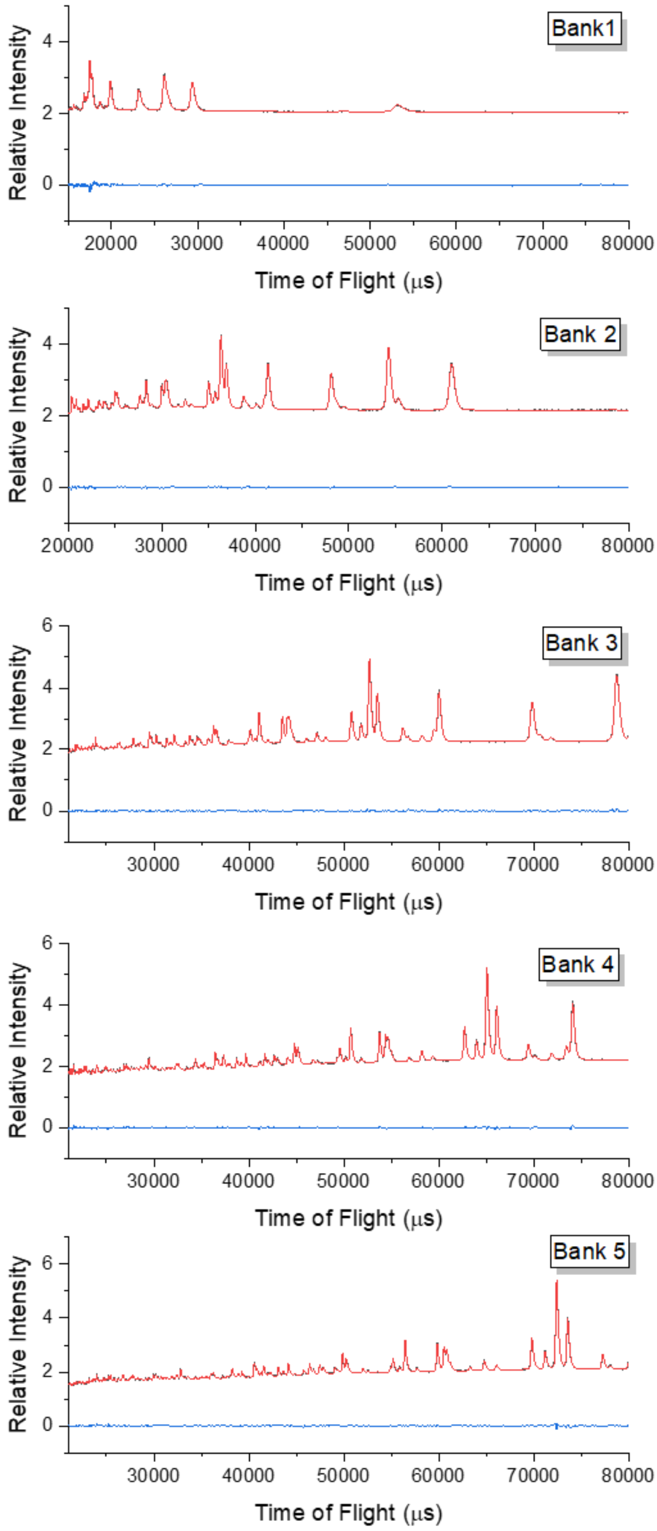

Figure S20. Neutron diffraction patterns and Rietveld refinement for $[CrC_8H_4O_5]{\cdot}3.67(D_2O)$

7. Inelastic neutron scattering

INS spectra were collected on the VISION spectrometer at Spallation Neutron Source, Oak Ridge National Laboratory (USA) and on the TOSCA beamline at ISIS Neutron and Muon Source (U.K.). The sample of desolvated MFM-300(Al) was loaded into a cylindrical vanadium sample container with an indium vacuum seal and connected to a gas handling system. The sample was degassed at 403 K and 10^{-7} mbar for 24 h to remove any residual trace guest water molecules. The temperature during data collection was controlled using a closed cycle refrigerator (CCR) cryostat (10 ± 0.1 K). For the water-loaded samples, a known amount of water was loaded into the cell, which was then sealed and heated to 393 K and maintained at that temperature for 2 h before being cooled down to 10 K for INS data collection.

Modelling by Density Functional Theory (DFT) of the bare and H_2O-loaded MFM-300(Al) was performed using the Vienna Ab initio Simulation Package (VASP).[13] The calculation used the Projector Augmented Wave (PAW) method[14,15] to describe the effects of core electrons, and Perdew-Burke-Ernzerhof (PBE)[16] implementation of the Generalized Gradient Approximation (GGA) for the exchange-correlation functional. Energy cut-off was 800 eV for the plane-wave basis of the valence electrons. The lattice parameters and atomic coordinates determined by neutron powder diffraction in this work were used as the initial structure. The electronic structure was calculated on the Γ-point for the unit cell (144 atoms for the blank MOF). The total energy tolerance for electronic energy minimization was 10^{-8} eV, and for structure optimization it was 10^{-7} eV. The maximum interatomic force after relaxation was below 0.001 eV/Å, and the optB86b-vdW functional for dispersion corrections was applied.[17] The vibrational eigen-frequencies and modes were then calculated by solving the force constants and dynamical matrix using Phonopy.[18] The OClimax software was used to convert the DFT-calculated phonon results to the simulated INS spectra.[19]

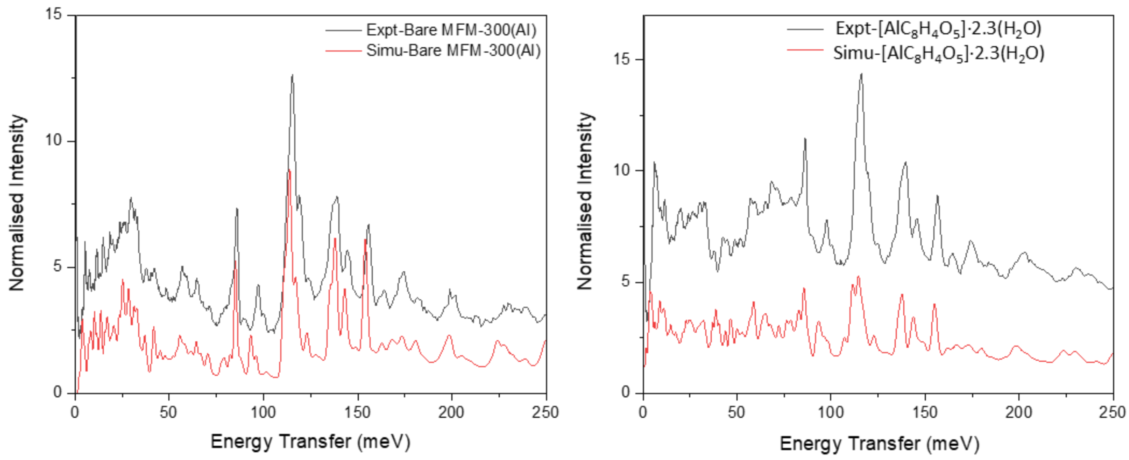

Figure S21. Comparison of experimental and simulated INS spectra of bare and H_2O-loaded MFM-300(Al).

8. Solid state nuclear magnetic resonance

^{1}H-pulses of 100 kHz were used for excitation and SPINAL-64 heteronuclear decoupling[20] using ^{27}Al-pulses of 0.5 μs duration (small flip angle with radio frequency field amplitude of ~70 kHz) were employed for ^{27}Al direct excitation (DE)MAS experiments. ^{13}C-Pulses and spin-locking at 50 kHz were used for {^{1}H-}^{13}C CPMAS experiments with corresponding ramped (70-100 %) ^{1}H spin-locking at ~73 kHz (100%) for 2 ms. A Hahn-echo (τ_r–π–τ_r) sequence of 2 rotor periods (τ_r) total duration was applied to ^{13}C after CP to circumvent receiver dead-time. For the ^{1}H-^{27}Al 2D CP (HETCOR) dipolar correlation experiments, 500 μs of CP spin-locking was applied with fixed-amplitude RF irradiation of ~28 kHz for ^{1}H and ~4 kHz for ^{27}Al. 26 complex t_1 increments were acquired with an indirect dimension dwell time of 83.33 μs. For static ^{2}H NMR spectroscopy, a quadrupolar echo ($\pi/2 - \tau - \pi/2 - \tau$) sequence was used with 4.5 μs pulses ($\pi/2$) and 10 μs delays (τ). Spectral deconvolution and peak fitting were performed in the solid line-shape analysis (SOLA) module v2.2.4 in Bruker TopSpin v4.0.9. ^{1}H and ^{13}C chemical shifts are given with respect to TMS (0 ppm) and ^{27}Al and ^{2}H NMR used the same magnetic field setting.

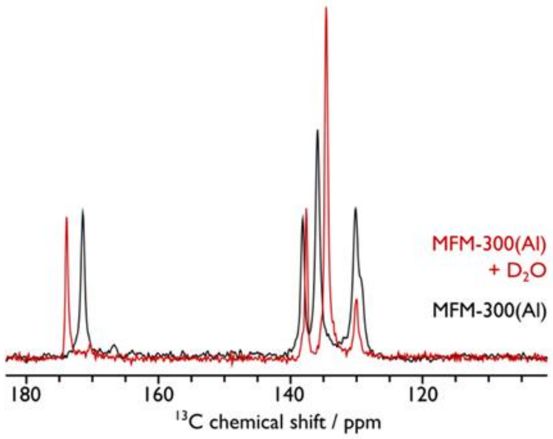

Figure S22. {^{1}H-}^{13}C CPMAS NMR spectra of D_2O-saturated (red) and pristine (black) MFM-300(Al), recorded with a MAS frequency of 12 kHz under ambient conditions.

9. Attenuated total reflectance FT infrared spectroscopy.

We carried out ATR-FTIR experiments with dry and wet samples of MFM-300(Al). ATR-FTIR spectra were collected for freshly activated sample, and for samples kept at RH = 10% and 99%, 25° C overnight. According to the adsorption isotherm of water at 25° C with MFM-300(Al), the water uptake at RH = 10% is 0.02 g/g. This corresponds to the chemical formula: $[AlC_8H_4O_5]\cdot 0.23(H_2O)$, which is close to the low loading sample in the NPD experiment. From the ATR-FTIR spectra, the -OH stretching band (3693 cm^{-1}) of the bridging hydroxyl along the AlO_6 chains of the activated sample and the sample kept at RH=10% showed negligible differences, further confirming the absence of strong interaction between adsorbed water and the bridging -OH group at low loading.

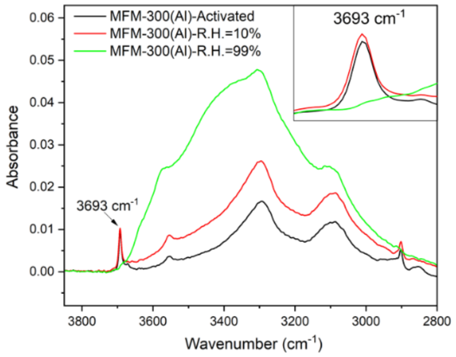

Figure S23. ATR-FTIR spectra of dry and wet samples of MFM-300(Al).

10. SEM images of MFM-300(M) (M = Al, Fe, Cr, V).

The morphology of MFM-300(Al, Fe, Cr, V) was examined using field emission scanning electron microscopy (FESEM, Hitachi SU-8010) with an acceleration voltage of 10 kV. The samples were mounted onto machined silicon stubs, utilizing carbon adhesive tabs for secure attachment. Imaging was carried out with a working distance of 7.8 mm.

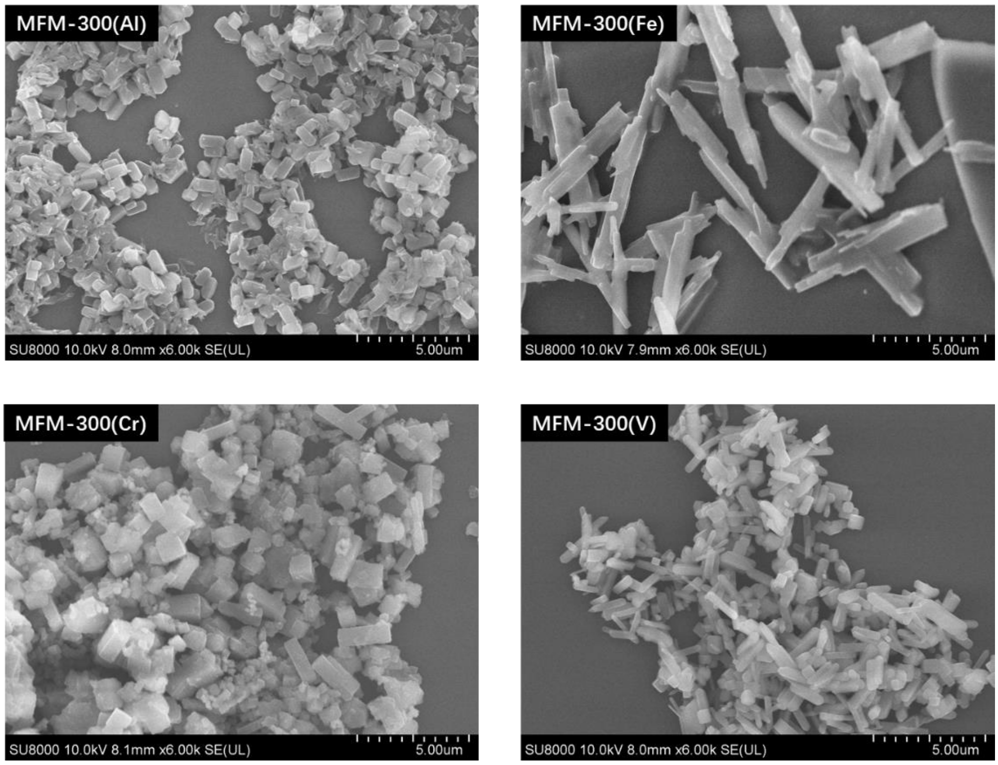

Figure S24. SEM images of MFM-300(M) (M = Al, Fe, Cr, V).

References

(1) Lin, X.; Telepeni, I.; Blake, A. J.; Dailly, A.; Brown, C. M.; Simmons, J. M.; Zoppi, M.; Walker, G. S.; Thomas, K. M.; Mays, T. J.; Hubberstey, P.; Champness, N. R.; Schröder, M. High Capacity Hydrogen Adsorption in Cu(II) Tetracarboxylate Framework Materials: The Role of Pore Size, Ligand Functionalization, and Exposed Metal Sites. *J. Am. Chem. Soc.* **2009**, *131*, 2159–2171.

(2) Yang, S.; Sun, J.; Ramirez-Cuesta, A. J.; Callear, S. K.; David, W. I. F.; Anderson, D. P.; Newby, R.; Blake, A. J.; Parker, J. E.; Tang, C. C.; Schröder, M. Selectivity and Direct Visualization of Carbon Dioxide and Sulfur Dioxide in a Decorated Porous Host. *Nat. Chem.* **2012**, *4*, 887–894.

(3) Zhang, X.; Da Silva, I.; Godfrey, H. G. W.; Callear, S. K.; Sapchenko, S. A.; Cheng, Y.; Vitórica-Yrezábal, I.; Frogley, M. D.; Cinque, G.; Tang, C. C.; Giacobbe, C.; Dejoie, C.; Rudić, S.; Ramirez-Cuesta, A. J.; Denecke, M. A.; Yang, S.; Schröder, M. Confinement of Iodine Molecules into Triple-Helical Chains within Robust Metal-Organic Frameworks. *J. Am. Chem. Soc.* **2017**, *139*, 16289–16296.

(4) Han, X.; Lu, W.; Chen, Y.; Da Silva, I.; Li, J.; Lin, L.; Li, W.; Sheveleva, A. M.; Godfrey, H. G. W.; Lu, Z.; Tuna, F.; McInnes, E. J. L.; Cheng, Y.; Daemen, L. L.; Mcpherson, L. J. M. C.; Teat, S. J.; Frogley, M. D.; Rudić, S.; Manuel, P.; Ramirez-Cuesta, A. J.; Yang, S.; Schröder, M. High Ammonia Adsorption in MFM-300 Materials: Dynamics and Charge Transfer in Host-Guest Binding. *J. Am. Chem. Soc.* **2021**, *143*, 3153–3161.

(5) Nuhnen, A.; Janiak, C. A Practical Guide to Calculate the Isosteric Heat/Enthalpy of Adsorption: Via Adsorption Isotherms in Metal-Organic Frameworks, MOFs. *Dalton Trans.* **2020**, *49*, 10295 – 10307.

(6) Matemb Ma Ntep, T. J.; Wahiduzzaman, M.; Laurenz, E.; Cornu, I.; Mouchaham, G.; Dovgaliuk, I.; Nandi, S.; Knop, K.; Jansen, C.; Nouar, F.; Florian, P.; Füldner, G.; Maurin, G.; Janiak, C.; Serre, C. When Polymorphism in Metal–Organic Frameworks Enables Water Sorption Profile Tunability for Enhancing Heat Allocation and Water Harvesting Performance. *Adv. Mater.* **2023**. *36*, 2211302.

(7) De Lange, M. F.; Verouden, K. J. F. M.; Vlugt, T. J. H.; Gascon, J.; Kapteijn, F. Adsorption-Driven Heat Pumps: The Potential of Metal-Organic Frameworks. *Chem. Rev.* **2015**, *115*, 12205–12250.

(8) Zhang, B.; Zhu, Z.; Wang, X.; Liu, X.; Kapteijn, F. Water Adsorption in MOFs: Structures and Applications. *Adv. Funct. Mater.* **2024**, *34*, 2304788.

(9) Cho, K. H.; Borges, D. D.; Lee, U. H.; Lee, J. S.; Yoon, J. W.; Cho, S. J.; Park, J.; Lombardo, W.; Moon, D.; Sapienza, A.; Maurin, G.; Chang, J. S. Rational Design of a Robust Aluminum Metal-Organic Framework for Multi-Purpose Water-Sorption-Driven Heat Allocations. *Nat. Commun.* **2020**, *11*, 11–18.

(10) Truong, B. N.; Borges, D. D.; Park, J.; Lee, J. S.; Jo, D.; Chang, J.; Cho, S. J.; Maurin, G.; Cho, K. H.; Lee, U. Tuning Hydrophilicity of Aluminum MOFs by a Mixed-Linker Strategy for Enhanced Performance in Water Adsorption-Driven Heat Allocation Application. *Adv. Sci.* **2023**, *10*, 202301311.

(11) Liu, Z.; Xu, J.; Xu, M.; Huang, C.; Wang, R.; Li, T.; Huai, X. Ultralow-Temperature-Driven Water-Based Sorption Refrigeration Enabled by Low-Cost Zeolite-like Porous Aluminophosphate. *Nat. Commun.* **2022**, *13*, 193.

(12) Coelho, A. A. TOPAS and TOPAS-Academic: An Optimization Program Integrating Computer Algebra and Crystallographic Objects Written in C++: An. *J. Appl. Crystallogr.* **2018**, *5*, 210–218.

(13) Kresse, G.; Furthmüller, J. Efficient Iterative Schemes for Ab Initio Total-Energy Calculations Using a Plane-Wave Basis Set. *Phys. Rev. B.* **1996**, *54*, 11169–11186.

(14) Kresse, G.; Joubert, D. From Ultrasoft Pseudopotentials to the Projector Augmented-Wave Method. *Phys. Rev. B.* **1999**, *59*, 1758–1775.

(15) Blöchl, P. E. Projector Augmented-Wave Method. *Phys. Rev. B.* **1994**, *50*, 17953–17979.

(16) Perdew, J. P.; Burke, K.; Ernzerhof, M. Generalized Gradient Approximation Made Simple. *Phys. Rev. Lett.* **1996**, *77*, 3865–3868.

(17) Klimeš, J.; Bowler, D. R.; Michaelides, A. Chemical Accuracy for the van Der Waals Density Functional. *J. Phys. Condens. Matter* **2010**, *22*, 022201.

(18) Togo, A.; Tanaka, I. First Principles Phonon Calculations in Materials Science. *Scr. Mater.* **2015**, *108*, 1–5.

(19) Cheng, Y. Q.; Daemen, L. L.; Kolesnikov, A. I.; Ramirez-Cuesta, A. J. Simulation of Inelastic Neutron Scattering Spectra Using OCLIMAX. *J. Chem. Theory. Comput.* **2019**, *15*, 1974–1982.

(20) Bräuniger, T.; Wormald, P.; Hodgkinson, P. Improved Proton Decoupling in NMR Spectroscopy of Crystalline Solids Using the SPINAL-64 Sequence. *Monatsh. Chem.* **2002**, *133*, 1549–1554.
